# A Degradable Nanoplatform Integrating Glucose Oxidase and Copper Selenide for Near-Infrared-Enhanced Multimodal Synergistic Therapy in Lung Cancer

**DOI:** 10.34133/bmr.0404

**Published:** 2026-08-14

**Authors:** Yong Zhang, Lei Xue, Lin Shi, Mengni Zhu, Jun Li, Degang Liu, Qiang Yang, Jiebai Zhou, Zilong Liu, Dawei Yang, Jiangzhou Peng

**Affiliations:** ^1^ Department of Pulmonary, Zhongshan Hospital, Fudan University, Shanghai 200032, China.; ^2^ Department of Thoracic Surgery, The Third Affiliated Hospital of Southern Medical University, Guangzhou 510630, China.; ^3^ Shanghai Key Laboratory of Magnetic Resonance, School of Physics and Electronic Science, East China Normal University, Shanghai 200241, China.

## Abstract

Lung cancer remains a leading cause of cancer-related mortality worldwide, with therapeutic outcomes frequently constrained by drug resistance, tumor heterogeneity, and systemic toxicity. Multimodal synergistic therapy has emerged as a promising strategy to address these challenges. In this study, we developed a biodegradable nanosystem, designated CSZG, which integrates glucose oxidase (GOx) and copper-rich copper selenide (Cu_2−*x*_Se) within a ZIF-8 nanocarrier for lung cancer therapy. CSZG exhibited pH-responsive GOx release, favorable colloidal stability, and retained glucose-responsive catalytic activity of GOx. GOx supplied hydrogen peroxide (H_2_O_2_) through glucose oxidation, while Cu^+^ catalyzed the conversion of H_2_O_2_ into cytotoxic hydroxyl radicals (•OH) under near-infrared (NIR) irradiation, thereby enhancing chemodynamic therapy. Electron spin resonance analysis directly confirmed NIR-enhanced •OH generation, particularly under acidic conditions. In addition, CSZG + NIR induced copper-dependent mitochondrial dysfunction with cuproptosis-associated features, as evidenced by aggregation of dihydrolipoamide S-acetyltransferase, down-regulation of ferredoxin 1 , depletion of Fe–S cluster proteins, and partial reversal by tetrathiomolybdate. The Cu_2−*x*_Se-containing platform also promoted macrophage polarization toward an M1-like phenotype. In vivo, CSZG + NIR achieved a tumor inhibition rate of 91.7% and demonstrated favorable short-term systemic biosafety under the tested therapeutic conditions. Collectively, these findings highlight CSZG as a promising multimodal therapeutic platform for lung cancer.

## Introduction

Lung cancer persists as a leading cause of global cancer-related mortality. According to GLOBOCAN 2022, it accounts for approximately 18.7% of all cancer deaths [1,2]. Although chemotherapy, radiotherapy, immunotherapy, and nanomedicine-based strategies have been extensively explored [3–5], therapeutic outcomes remain frequently compromised by tumor heterogeneity, acquired resistance, and systemic toxicities—particularly in patients with advanced disease. Recent precision-activation nanomedicine designs aim to improve tumor selectivity, enhance efficacy, and reduce off-target effects [6–10].

Multimodal synergistic therapy has attracted increasing attention as a strategy to amplify therapeutic outcomes while mitigating adverse effects, especially when coupled to oxidative stress-related regulated cell death pathways exploitable in lung cancer [11,12]. Relative to single-modality platforms, multimodal designs integrate multiple functional agents to act concurrently at the tumor site, inducing cancer cell death through complementary mechanisms [13,14]. Prior work on nanoparticle-based drug delivery, inorganic/metal-containing nanomaterials, and porous nanocarriers has underscored their potential for improving drug loading, controlled release, therapeutic selectivity, and anticancer efficacy [15–21].

For instance, combining immunotherapy with chemodynamic therapy (CDT) holds potential for alleviating tumor immune evasion and the immunosuppressive microenvironment [22–24], while integrating chemotherapy with photodynamic therapy (PDT) or photothermal therapy (PTT) can improve treatment selectivity and enable visualization of drug delivery [25]. Nonetheless, challenges remain in optimizing mechanistic integration, synergistic precision, and delivery efficiency for such multimodal therapies [26–31].

**Fig. 1.**
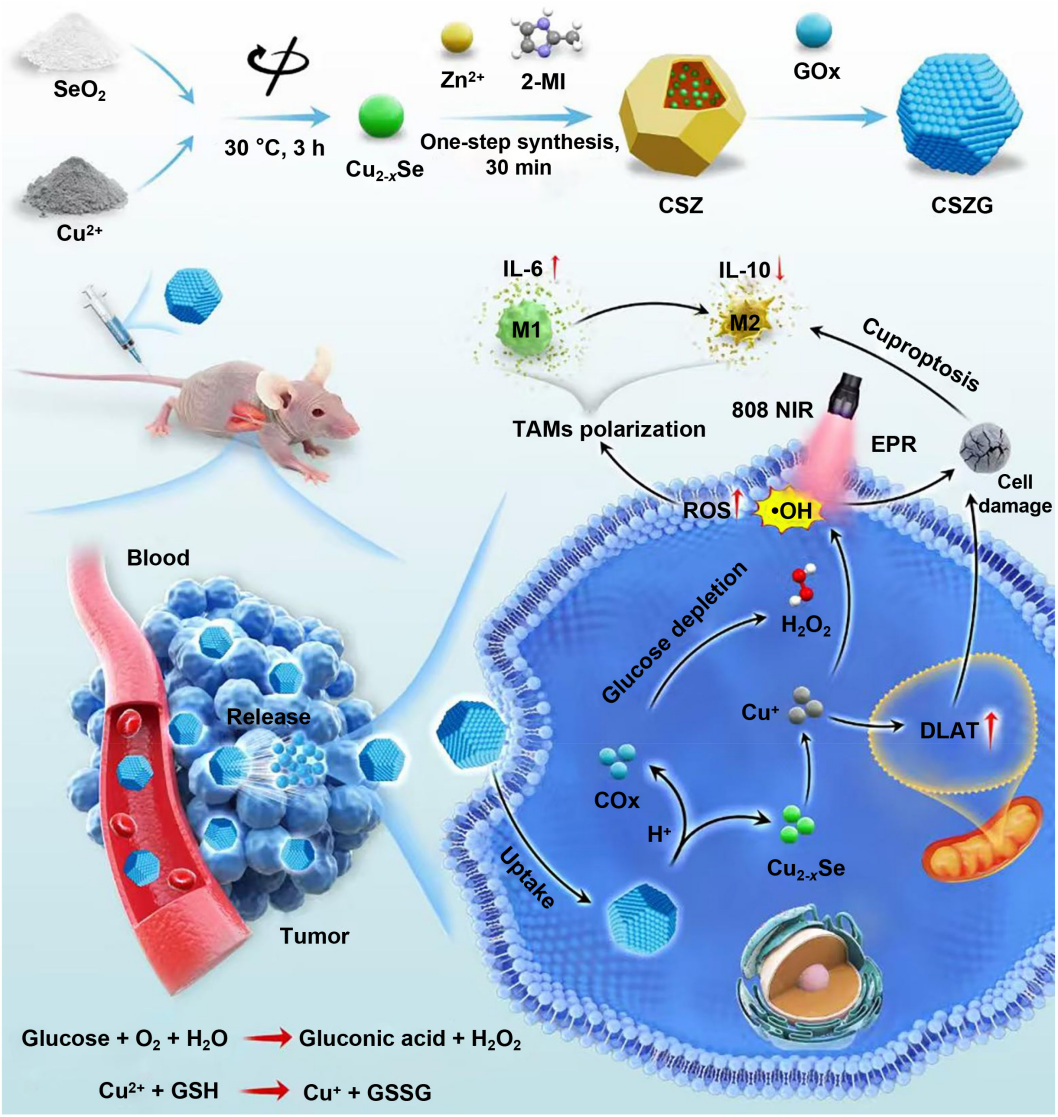
Schematic illustration of the synthesis of CSZG and its multimodal synergistic anticancer mechanism.

Based on these considerations, we constructed a degradable nanoplatform using zeolitic imidazolate framework-8 (ZIF-8) as a carrier to coload glucose oxidase (GOx) and Cu-rich Cu_2−*x*_Se nanoparticles, designated CSZG (Fig. 1). Cu_2−*x*_Se, synthesized under mild conditions, contains abundant Cu^+^ that exerts antitumor effects through 2 synergistic routes. First, Cu^+^ interacts with lipoylated tricarboxylic acid cycle proteins, promoting aggregation of dihydrolipoamide S-acetyltransferase (DLAT) and depleting Fe–S cluster proteins—events aligned with cuproptosis-associated mitochondrial stress. Second, Cu^+^ catalyzes H_2_O_2_-to-•OH conversion (Fenton-like CDT), amplified under near-infrared (NIR) irradiation by the photothermal property of Cu_2−*x*_Se. Beyond direct cytotoxicity, Cu_2−*x*_Se promotes macrophage repolarization toward an M1-like phenotype. GOx supplies H_2_O_2_ via glucose oxidation for Cu-mediated catalysis, while acid-labile ZIF-8 enables pH-responsive payload release and biodegradation in the acidic tumor microenvironment. This multifunctional nanosystem, through locally activated multimechanistic synergy, achieves markedly enhanced multimodal antitumor efficacy.

## Materials and Methods

### Materials

Cetrimonium bromide (CTAB), selenium dioxide (SeO_2_), vitamin C (VC), copper sulfate pentahydrate (CuSO_4_·5H_2_O), zinc nitrate hexahydrate (Zn(NO_3_)_2_·6H_2_O), 2-methylimidazole, and anhydrous D-glucose were purchased from Innochem (Beijing, China). GOx was from Bioisco (USA). Dimethyl sulfoxide and Annexin V-FITC/PI apoptosis kit were from Solarbio (Beijing, China). Interleukin-4 (IL-4) was from Beyotime (Shanghai, China). CCK-8 kit was from Zeta Life (USA). Enzyme-linked immunosorbent assay (ELISA) kits for LDH, CYFRA21-1, IL-10, and IL-6 were from Beyotime. Terminal deoxynucleotidyl transferase-mediated dUTP nick-end labeling (TUNEL) kit was from Servicebio (Wuhan, China). H_2_O_2_ was from Chengdu Jinshan. Horseradish peroxidase (HRP) was from Boer (Shanghai). 2′,7′-Dichlorodihydrofluorescein diacetate (DCFH-DA) was from Elabscience (Wuhan, China). 2,2′-Azino-bis(3-ethylbenzothiazoline-6-sulfonic acid) diammonium salt (ABTS) was from Sigma-Aldrich. All other reagents were of analytical grade and used as received.

### Synthesis of Cu_2−*x*_Se nanoparticles

Cu_2−*x*_Se was synthesized as follows: 1.6 ml of CTAB + 5.5 ml of deionized water were placed in a round-bottom flask; 0.1 ml of SeO_2_ and 0.3 ml of VC were added and vigorously stirred for 10 min. A mixed solution of 0.1 ml of CuSO_4_·5H_2_O + 0.4 ml of VC was then introduced. The reaction was maintained at 30 °C with stirring for 3 h until a homogeneous green solution formed. The product was dialyzed against deionized water for 24 h (water changed 6 times) to remove small-molecule impurities and stored at 4 °C.

### Synthesis of CSZ (Cu_2−*x*_Se@ZIF-8)

Two milliliters of Cu_2−*x*_Se (5 mg/ml) was mixed with 14 ml of Zn(NO_3_)_2_·6H_2_O (3.71 mg/ml) and stirred at room temperature (RT) for 1 h. Then, 14 ml of 2-methylimidazole (2.05 mg/ml) was added and stirred for 2 h. The product was termed CSZ.

### Synthesis of CSZG (Cu_2−*x*_Se@ZIF-8@GOx)

Cu_2−*x*_Se (2 ml) was added to a round-bottom flask containing 14 ml of Zn(NO_3_)_2_·6H_2_O, 14 ml of 2-methylimidazole, and GOx aqueous solution (0.5 mg/ml). The mixture was stirred at RT under N_2_ for 30 min. The resulting CSZG was washed 3 times with water, centrifuged (12,000 rpm, 2 min), and dried under vacuum.

### Characterizations

Ultraviolet–visible (UV–Vis) absorption spectra were recorded using a UV-2600 spectrophotometer (Shimadzu, Japan). Dynamic light scattering (DLS) measurements for particle size, polydispersity index (PDI), and zeta potential were performed using a BeNano 90 Zeta instrument (Bettersize Instruments, China). Transmission electron microscopy (TEM) images were obtained with an HT7700 microscope (Hitachi, Japan). Powder x-ray diffraction (XRD) patterns of Cu_2−*x*_Se, ZIF-8, CSZ, and CSZG were recorded on a Rigaku Ultima IV diffractometer (Rigaku, Japan). Fourier transform infrared (FTIR) spectra of Cu_2−*x*_Se, CSZ, and CSZG were acquired in the range of 500 to 4,000 cm^–1^ using a Nicolet 6700 spectrometer. X-ray photoelectron spectroscopy (XPS) of CSZG was performed using a Thermo Scientific K-Alpha system (USA).

For stability evaluation, the particle size and PDI of CSZG dispersions were monitored over 72 h at 25 °C in phosphate-buffered saline (PBS) or PBS containing 10% fetal bovine serum (FBS) using DLS. For pH-dependent degradation analysis, CSZG dispersions (1 mg/ml) were incubated in PBS at pH 6.5 or pH 7.4 at 37 °C. At predetermined time points (0, 6, 12, 24, 48, and 72 h), aliquots were collected, and changes in absorbance were monitored by UV–Vis spectrophotometry.

### GOx loading, encapsulation, and release analysis

GOx concentration was determined using a Bradford assay (Bio-Rad, USA) according to the manufacturer’s instructions. A standard curve was generated using GOx solutions of known concentrations (0 to 2 mg/ml) measured at 595 nm. To determine the encapsulation efficiency (EE) and loading efficiency (LE) of GOx in CSZG, the absorbance of the original GOx solution and the supernatant after nanoparticle preparation was measured. EE and LE were calculated as follows:

$$\begin{array}{c} \begin{array}{c} \begin{array}{c} \mathrm{EE}\;\left(\%\right)=\\ \left(\text{Amount of}\;\mathrm{GOx}\;\text{loaded in nanoparticles}/\text{Total amount of}\;\mathrm{GOx}\;\text{added}\right)\times 100\% \end{array} \end{array} \end{array}$$ (1)

$$\begin{array}{c} \mathrm{LE}\;\left(\%\right)=\\ \left(\text{Amount of}\;\mathrm{GOx}\;\text{loaded in nanoparticles}/\text{Total weight of nanoparticles}\right)\times 100\% \end{array}$$ (2)

The EE and LE of GOx in CSZG were determined to be 90% and 60%, respectively.

For the release study, 3.7 mg of CSZG was incubated in 20 ml of PBS at pH 6.0, 6.5, or 7.4 at 37 °C with gentle shaking (100 rpm). At specified time points (0, 1, 2, 4, 6, 8, 12, 16, 20, and 24 h), the suspension was centrifuged at 12,000 rpm for 2 min, and the supernatant was collected. The released GOx in the supernatant was quantified by the Bradford assay at 595 nm. The cumulative release profile was calculated as the percentage of GOx released relative to the total loaded amount.

### Cu^+^ release assay

Cu^+^ release from CSZG was measured using a neocuproine-based colorimetric assay. Briefly, Cu^+^ standard solutions were prepared from cuprous chloride and stabilized as [CuCl_2_]^–^ in a sodium chloride–hydrochloric acid medium under nitrogen protection. Samples were mixed with acetate–sodium acetate buffer to adjust the pH to approximately 5.5, followed by the addition of ammonium citrate to mask potential interfering metal ions. Neocuproine ethanol solution was then added, allowing Cu^+^ to form a stable yellow 1:2 complex with neocuproine. After incubation at RT for 5 min, the absorbance was measured at 454 nm using a reagent blank as the reference. For CSZG samples, Cu^+^ release was measured at different time points under pH 6.0 conditions.

### Assessment of oxygen generation in response to H_2_O_2_

Deionized water (2 ml, blank), Cu_2−*x*_Se solution (2 ml, 1 mg/ml), and CSZ solution (2 ml, 1 mg/ml) were each mixed with 8 ml of H_2_O_2_ solution (125 μM) and incubated for 5 h. The dissolved oxygen content in the solutions was measured using a JPBJ-608 dissolved oxygen meter (Leici, China).

### Enzyme activity assay

The catalytic activity of GOx after incorporation into CSZG was evaluated using an HRP/ABTS colorimetric assay. Briefly, PBS solutions (2 ml; 0.01 M, pH 7.4) containing HRP (0.05 mg/ml), ABTS (274 μg/ml), and different concentrations of glucose were prepared. Free GOx (0.2 μg) or CSZG (2 μg) was then added to each solution. GOx catalyzes the oxidation of glucose to generate H_2_O_2_, which subsequently oxidizes ABTS in the presence of HRP, producing a colored product detectable at 415 nm. The absorbance at 415 nm was recorded to evaluate glucose-dependent H_2_O_2_ generation and the retained functional catalytic activity of GOx within CSZG.

### Detection of hydroxyl radicals (•OH)

The •OH-generating capacity of CSZG was evaluated using 3,3′,5,5′-tetramethylbenzidine (TMB) colorimetric and electron spin resonance (ESR) assays.

*TMB assay:* Different concentrations of CSZG (0, 25, 50, 100, 200, and 400 μg/ml) were incubated with 200 μM H_2_O_2_ in PBS for 10 min, followed by the addition of 1% TMB solution. UV–Vis absorbance spectra were recorded to evaluate the concentration-dependent catalytic oxidation of TMB. Photographs of the TMB solutions were taken at 0 and 10 min to visually record the color change. For NIR-enhanced •OH detection, CSZG (2 ml, 2 mg/ml in PBS) was incubated with 20 mM glucose, 10 mM TMB, and 5 mM glutathione under 808 nm laser irradiation (1 W/cm^2^) for 1 h. The solution was then centrifuged, and TMB stop solution (0.5 M HCl) was added to the supernatant. After 3 h of incubation, absorbance was measured at 450 nm.

*ESR assay:* ESR spectroscopy was performed using 5,5-dimethyl-1-pyrroline N-oxide (DMPO) as a spin-trapping agent to directly detect hydroxyl radical (•OH) generation. Briefly, DMPO solution (200 μl) was added to PBS or CSZG solution containing H_2_O_2_ (final volume 400 μl), and the samples were then loaded into capillary tubes for ESR analysis. The ESR groups included DMPO, DMPO + NIR, CSZG, and CSZG + NIR at pH 7.4, and CSZG + NIR at pH 6.5. For NIR-treated groups, samples were irradiated with an 808-nm laser at 1 W/cm^2^ for 1 h before ESR detection. The characteristic DMPO-•OH quartet signal was recorded to evaluate •OH generation under NIR irradiation and acidic conditions.

### Cell culture and animals

A549, H460, and BV2 cells were obtained from the American Type Culture Collection. Dulbecco’s Modified Eagle Medium (DMEM), FBS, penicillin–streptomycin solution, and 0.25% trypsin-EDTA were purchased from Gibco. Cells were cultured in a humidified incubator at 37 °C with 5% CO_2_.

Balb/c nude mice and Balb/c mice (18 to 22 g, 6 to 8 weeks old, male) were obtained from Zhuhai Bestest Bio-Tech Co., Ltd. (Zhuhai, China). All animals were housed under standard laboratory conditions with controlled temperature and humidity and free access to food and water. All animal experiments were approved by the Ethics Committee of Zhongshan Hospital Affiliated to Fudan University and conducted in accordance with relevant guidelines.

### Cell viability assay

Cells were seeded in 96-well plates (1 × 10^4^ cells/well) and adhered overnight. Medium was replaced with serial Cu_2−*x*_Se/CSZ/CSZG (0 to 200 μg/ml) in complete DMEM, 24 h incubation. CCK-8 reagent was added (10 μl/well), 1 h RT, 450 nm read. Cell viability was calculated using the following formula: cell viability (%) = (*A*_450_ sample/*A*_450_ control) × 100, where *A*_450_ sample and *A*_450_ control represent the absorbance of treated and untreated wells, respectively.

### Cell apoptosis assay

A549 and H460 cells (5 × 10^4^ cells/well) were seeded in 24-well plates and cultured overnight. The cells were then divided into 7 groups: PBS, NIR, ZIF-8, ZIF-8@GOx, Cu_2−*x*_Se@ZIF-8 (CSZ), CSZG, and CSZG + NIR. These formulation-matched controls were designed to distinguish the effects of NIR irradiation alone, the ZIF-8 carrier, GOx loading, Cu_2−*x*_Se incorporation, and the complete CSZG + NIR treatment on tumor cell apoptosis. The concentration of each nanomaterial was 100 μg/ml. For laser treatment, cells were exposed to an 808-nm laser (0.1 W/cm^2^, 5 min) and then further incubated for 4 h in the dark. Subsequently, cells were washed 3 times with PBS, harvested using EDTA-free trypsin, and collected by centrifugation. The cell pellets were resuspended in binding buffer and stained with Annexin V-FITC and propidium iodide (PI, 5 μl each) for 10 min. Apoptosis was analyzed by flow cytometry. The total apoptotic rate was calculated as the sum of early and late apoptotic cell populations.

### Cellular uptake

A549 and H460 cells (1 × 10^5^ cells/well) were seeded in 12-well plates and cultured overnight. The medium was replaced with fresh DMEM containing 100 μg/ml of Rhodamine 6G (R6G)-labeled CSZG. After incubation for 3 or 6 h, cells were washed with PBS, fixed with 4% paraformaldehyde for 15 min, and stained. Nuclei were stained with 4′,6-diamidino-2-phenylindole (DAPI), and cell membranes were stained with DiO for 10 min. After washing, cellular uptake was visualized using a confocal laser scanning microscope (CLSM; Leica, Germany). For quantitative analysis, BV2 cells (5 × 10^5^ cells/well) were seeded in 6-well plates; incubated with 100 μg/ml of R6G-labeled CSZG for 2, 4, 6, or 8 h; washed; resuspended in PBS; and analyzed by flow cytometry to determine the mean fluorescence intensity (MFI) of R6G.

### Intracellular ROS detection

A549 and H460 cells (5 × 10^4^ cells/well) were seeded in 24-well plates and allowed to adhere overnight. Cells were randomly assigned to 7 groups: PBS, NIR, ZIF-8, ZIF-8@GOx, CSZ, CSZG, and CSZG + NIR. These groups enabled comparison of ROS generation associated with the carrier, GOx-mediated H_2_O_2_ self-supply, Cu_2−*x*_Se-mediated catalytic activity, and NIR-enhanced CSZG treatment. The nanomaterial concentration was 100 μg/ml. NIR-treated groups were exposed to an 808-nm laser (0.1 W/cm^2^, 5 min) followed by 4 h of incubation. Cells were then harvested, incubated with 10 μM DCFH-DA according to the manufacturer’s protocol, and intracellular ROS-associated fluorescence was quantified by flow cytometry.

### Immunofluorescence staining for DLAT and FDX1

A549/H460 cells were cultured on coverslips and treated as indicated. At 24 h posttreatment, cells were fixed with 4% paraformaldehyde (PFA) for 15 min, permeabilized with 0.1% Triton X-100 for 10 min, and blocked with 5% bovine serum albumin (BSA) for 30 min. Cells were then incubated overnight at 4 °C with anti-FDX1 and anti-DLAT primary antibodies (1:200 each), followed by Alexa Fluor-conjugated secondary antibodies (1:500) for 1 h at room temperature. Nuclei were counterstained with DAPI. Fluorescence images were acquired using a Leica microscope. At least three random fields per group were analyzed, and the mean fluorescence intensity (MFI) per cell was quantified using ImageJ.

### Western blot analysis of cuproptosis-associated mitochondrial proteins and TTM rescue assay

A549 and H460 cells were seeded in 6-well plates and cultured overnight until they reached approximately 60% to 80% confluence. To evaluate cuproptosis-associated mitochondrial protein alterations, cells were treated with PBS, NIR, ZIF-8, ZIF-8@GOx, CSZ, CSZG, or CSZG + NIR. The final concentration of each nanomaterial was 100 μg/ml. For NIR-treated groups, cells were incubated with the corresponding formulations for 4 h and then irradiated with an 808-nm laser at 0.1 W/cm^2^ for 5 min. Cells were further cultured until the total treatment time reached 24 h.

For copper-chelator rescue experiments, cells were divided into PBS, CSZG + NIR, tetrathiomolybdate (TTM), and CSZG + NIR + TTM groups. In the rescue group, cells were pretreated with TTM (10 μM) for 2 h before CSZG incubation and NIR irradiation as described above.

After treatment, total proteins were extracted using radioimmunoprecipitation assay (RIPA) lysis buffer containing protease inhibitors, and protein concentrations were determined using a bicinchoninic acid assay. Equal amounts of protein were separated by sodium dodecyl sulfate–polyacrylamide gel electrophoresis and transferred onto polyvinylidene fluoride membranes. After blocking with 5% nonfat milk, the membranes were incubated overnight at 4 °C with primary antibodies against FDX1, DLAT, NDUFS1, SDHB, and β-actin. The membranes were then incubated with HRP-conjugated secondary antibodies. Protein bands were visualized using enhanced chemiluminescence and quantified using ImageJ software. Relative protein expression levels were normalized to β-actin. All experiments were independently repeated at least 3 times.

### Macrophage repolarization assay

BV2 cells were used as macrophage-like cells to evaluate the immunomodulatory effect of the nanoplatform. Cells were seeded in 6-well plates at a density of 5 × 10^5^ cells/well and stimulated with murine IL-4 (20 ng/ml) for 24 h to induce an M2-like phenotype. The cells were then divided into 7 groups: PBS, NIR, ZIF-8, ZIF-8@GOx, CSZ, CSZG, and CSZG + NIR. These groups were used to evaluate the individual and combined effects of the ZIF-8 carrier, GOx loading, Cu_2−*x*_Se incorporation, and NIR irradiation on macrophage-related immune polarization. The nanomaterial concentration was 100 μg/ml. NIR-treated groups were exposed to an 808-nm laser(0.1 W/cm^2^, 5 min). After 24 h, cells were harvested and costained with CD206-APC and CD86-PE antibodies for flow cytometric analysis. Culture supernatants were collected for ELISA analysis of IL-6 and IL-10 levels. Total protein was extracted using RIPA buffer, and the expression levels of M1-associated markers (CD80 and CD86) and M2-associated markers (CD163 and CD206) were analyzed by Western blotting.

### In vivo biosafety evaluation

Balb/c mice were intravenously injected via the tail vein with different concentrations of CSZG or PBS (control). Animals were observed continuously for 24 h to monitor behavioral changes and survival. Subsequently, mice were euthanized, and major organs (heart, liver, spleen, lungs, and kidneys) were harvested for histological analysis (hematoxylin and eosin [H&E] staining).

### Hemolysis test

Fresh blood was collected from healthy mice, and red blood cells (RBCs) were obtained by centrifugation to remove plasma. RBCs were then incubated with different concentrations of CSZ or CSZG at 37 °C for 3 h. After incubation, the mixtures were centrifuged, and the absorbance of the supernatant was measured at 540 nm. PBS served as the negative control, and deionized water was used as the positive control. The hemolysis rate was calculated as follows: Hemolysis (%) = (Abs. of experimental group – Abs. of negative control)/(Abs. of positive control – Abs. of negative control) × 100%.

### Animal model establishment

A549 cell suspension (100 μl, 2 × 10^7^ cells/ml) was subcutaneously injected into the right axillary fat pad of Balb/c nude mice to establish a lung cancer xenograft model. The model was considered successfully established when the tumor volume reached 150 to 200 mm^3^. Tumor volume was calculated using the formula: Volume (mm^3^) = length × width^2^ × 0.5.

### In vivo metabolism and targeting

Following tumor model establishment, mice were randomly divided into 3 groups (*n* = 5 per group) and intravenously injected with indocyanine green (ICG), CSZ/ICG, or CSZG/ICG. Fluorescence imaging was performed using an IVIS Lumina II system (excitation: 740 nm, exposure time: 5 s) at 0, 0.5, 1, 3, 6, 12, 24, and 48 h postinjection. After the final imaging time point, mice were euthanized, and tumors and major organs were harvested for ex vivo fluorescence imaging and biodistribution analysis. Fluorescence intensities in tumors and major organs were quantified by region-of-interest analysis using Living Image software and normalized to the background signal and tissue area.

### In vivo antitumor efficacy evaluation

After the tumor volume reached 150 to 200 mm^3^, tumor-bearing mice were randomly divided into 8 groups (*n* = 5 per group): (a) PBS, (b) NIR, (c) ZIF-8, (d) ZIF-8@GOx, (e) CSZ, (f) CSZ + NIR, (g) CSZG, and (h) CSZG + NIR. These groups were designed to distinguish the individual and combined contributions of NIR irradiation alone, the ZIF-8 carrier, GOx loading, Cu_2−*x*_Se incorporation, NIR-enhanced CSZ, and the complete CSZG + NIR treatment. Each group received intravenous injections via the tail vein on days 0, 2, and 4, with 100 μl of the corresponding formulation (CSZ at 3.2 mg/kg; CSZG at 5 mg/kg). For NIR-treated groups, 808-nm laser irradiation (0.6 W/cm^2^) was performed 20 to 24 h after each injection for 5 min, followed by a 2-min interval and a second 5-min irradiation. Body weight and tumor volume were recorded every other day.

On day 12, all mice were sacrificed. Tumors were excised, weighed, photographed, and collected along with major organs for H&E staining, immunohistochemistry, and immunofluorescence (IF) analyses, including TUNEL, CD31, and Ki67 staining (performed by Servicebio, Wuhan, China). Quantitative analysis of TUNEL- and CD31-positive fluorescence signals was performed using ImageJ software from randomly selected tumor fields. For each tumor section, at least 3 randomly selected fields were analyzed. Blood samples were collected for serum biochemical analysis, including alanine aminotransferase (ALT), aspartate aminotransferase (AST), urea (UREA), and creatinine (CREA), to evaluate hepatic and renal function. Serum levels of LDH, CYFRA21-1, IL-6, and IL-10 were quantified using ELISA. Additionally, spleens were harvested to prepare single-cell suspensions, and the proportions of CD4^+^ and CD8^+^ lymphocytes were analyzed by flow cytometry.

### Tumor-associated macrophage repolarization and cuproptosis assessment

Tumor-associated macrophage (TAM) phenotypes were evaluated by IF staining and Western blot analysis. The expression levels of the M1 marker CD86 and the M2 marker CD206 were measured to assess macrophage polarization. Quantitative fluorescence analysis of CD86- and CD206-positive signals was performed using ImageJ software from at least 3 randomly selected tumor fields per section.

For flow cytometric analysis, fresh tumor tissues collected from the 8 treatment groups were placed in ice-cold PBS, and necrotic tissue, capsules, and surrounding adipose tissue were removed. Tumors were minced into approximately 1- to 2-mm^3^ fragments and digested in RPMI 1640 or DMEM containing collagenase IV, DNase I, and FBS at 37 °C for 30 to 45 min with gentle shaking. The digestion was terminated with complete medium, and the cell suspension was filtered through a 70-μm cell strainer. After centrifugation, RBCs were lysed with ACK lysis buffer when necessary, and the cells were washed and resuspended in FACS buffer.

Approximately 1 × 10^6^ cells were blocked with an Fc receptor blocking reagent and then stained with fluorophore-conjugated antibodies against CD11b, CD86, and CD206 at 4 °C in the dark. Blank, single-staining, and fluorescence-minus-one controls were used for compensation and gating. After washing, cells were resuspended in FACS buffer, filtered through a 40-μm cell strainer, and analyzed by flow cytometry. Cells were first gated based on FSC-A/SSC-A to exclude debris, followed by singlet selection to exclude doublets and aggregates. CD11b^+^ tumor-infiltrating myeloid/macrophage-enriched cells were then gated, and the proportions of CD86^+^ and CD206^+^ cells within the CD11b^+^ population were analyzed. Data were analyzed using FlowJo software.

Cuproptosis was analyzed by detecting the expression of key related proteins, including DLAT and FDX1, in tumor tissues via Western blot and/or IF.

### Statistical analysis

All quantitative data are presented as mean ± SD. Unless otherwise stated, in vitro experiments were independently repeated 3 times. For in vivo therapeutic studies, tumor growth, body weight, tumor weight, and tumor inhibition rate were analyzed with *n* = 5 mice per group. Sample sizes for other ex vivo analyses, including Western blotting, flow cytometry, ELISA, serum biochemical analysis, and IF quantification, are indicated in the corresponding figure legends (*n* = 3). Image-based quantification and Western blot densitometry were performed using ImageJ software, while flow cytometry data were analyzed using FlowJo software.

Comparisons between 2 groups were performed using an unpaired 2-tailed Student *t* test. Comparisons among more than 2 groups were analyzed using one-way analysis of variance (ANOVA) followed by Tukey’s post hoc test. Tumor growth and body weight curves measured over time were analyzed using 2-way ANOVA followed by Tukey’s multiple-comparison test. Statistical analyses were performed using GraphPad Prism 10.1.2 software. Statistical significance was defined as follows: *P < 0.05, **P < 0.01, ***P < 0.001, and ****P < 0.0001; ns, not significant.

## Results

### Preparation and characterization of CSZG

**Fig. 2.**
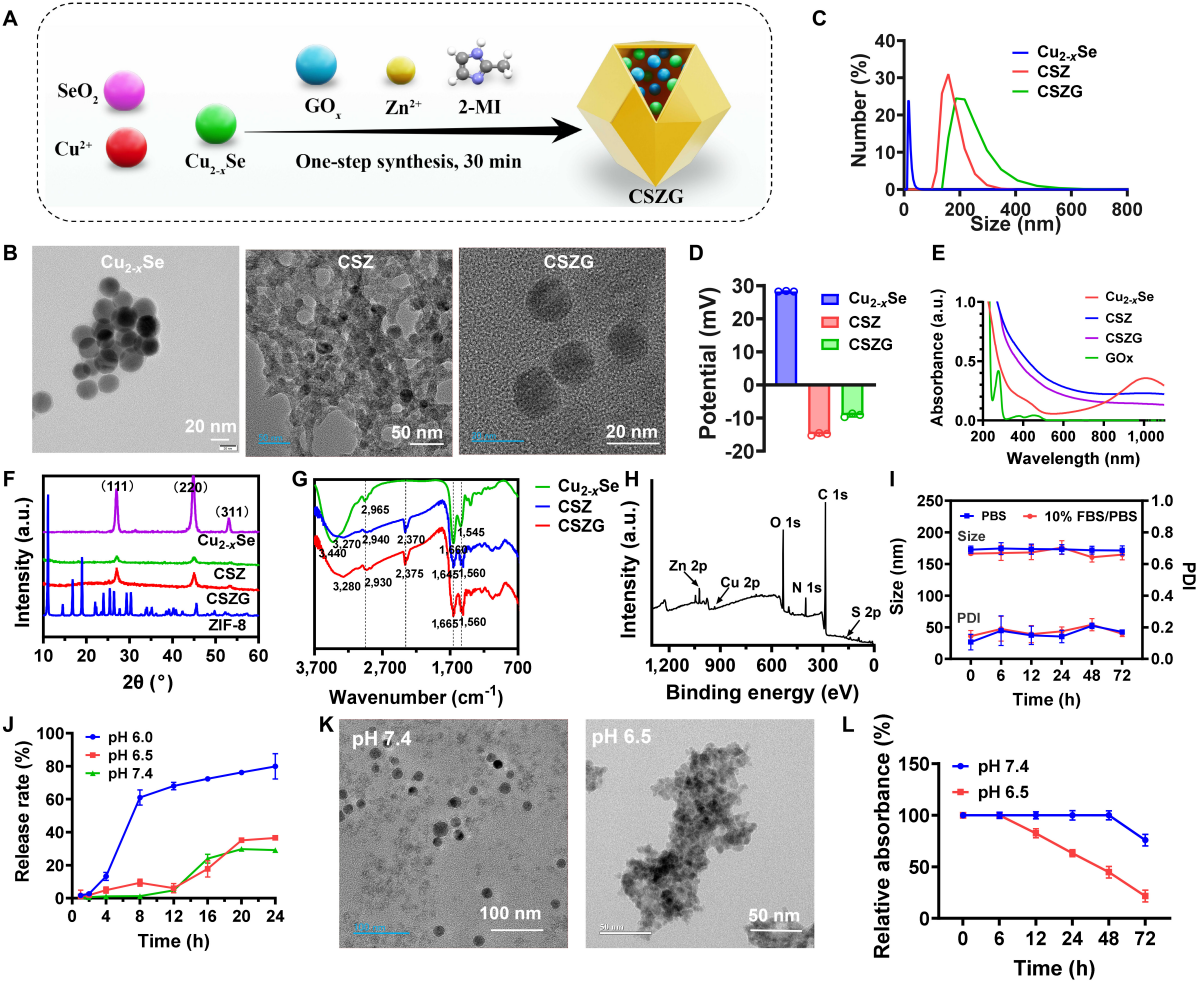
Characterization of CSZG nanoparticles. (A) Schematic illustration of the synthesis of CSZG via self-assembly of Cu_2−*x*_Se, GOx, Zn^2+^, and 2-methylimidazole. (B) TEM images of Cu_2−*x*_Se, CSZ, and CSZG (scale bars: 50 nm). (C) Particle size distribution of Cu_2−*x*_Se, CSZ, and CSZG measured by DLS. (D) Zeta potentials of Cu_2−*x*_Se, CSZ, and CSZG. (E) UV–Vis absorption spectra of Cu_2−*x*_Se, CSZ, CSZG, and free GOx. (F) XRD patterns of ZIF-8, Cu_2−*x*_Se, CSZ, and CSZG. (G) FTIR spectra of Cu_2−*x*_Se, CSZ, and CSZG. (H) XPS survey spectrum of CSZG showing the presence of Cu, Se, Zn, O, N, and C. (I) Hydrodynamic size of CSZG in PBS and PBS containing 10% FBS over 72 h, indicating colloidal stability. (J) Cumulative release profiles of GOx from CSZG at pH 6.0, 6.5, and 7.4 over 24 h. (K) TEM images of CSZG after incubation at pH 7.4 and pH 6.5 for 24 h, showing pH-responsive degradation. (L) Time-dependent relative absorbance of CSZG at 290 nm in PBS at pH 7.4 and pH 6.5 over 72 h. Absorbance values were normalized to the initial value at 0 h. Data are presented as mean ± SD (*n* = 3).

Cu_2−*x*_Se was synthesized and incorporated into ZIF-8 together with GOx in a one-pot self-assembly to yield CSZG (Fig. 2A). TEM showed relatively uniform, well-dispersed Cu_2−*x*_Se; after incorporation, CSZ exhibited slight aggregation, whereas CSZG retained a generally spherical morphology (Fig. 2B). DLS gave hydrodynamic diameters of ~19 nm (Cu_2−*x*_Se), 169 nm (CSZ), and 180 nm (CSZG), indicating that GOx loading did not cause a marked size increase (Fig. 2C). ζ-potentials were +28.3 mV (Cu_2−*x*_Se), –14.8 mV (CSZ), and –9.2 mV (CSZG) (Fig. 2D). UV–Vis showed the characteristic GOx band at 276 nm in both free GOx and CSZG (Fig. 2E), confirming GOx incorporation. Based on a GOx calibration curve (Fig. S1A), EE and LE of GOx in CSZG were 90% and 60%, respectively.

XRD of pristine ZIF-8 showed sharp characteristic peaks, which disappeared upon Cu_2−*x*_Se incorporation (CSZ), indicating framework collapse/reconstruction into a predominantly amorphous/disordered structure. CSZG gave a similar profile to CSZ, suggesting that GOx loading introduced no new crystalline phase (Fig. 2F). FTIR further supported composite formation (Fig. 2G). XPS confirmed Cu, Se, and Zn in CSZG, with elevated O/N consistent with surface-associated GOx (Fig. 2H). High-resolution XPS: Cu 2p consistent with low-valence Cu (Cu^+^/Cu^0^) from Cu_2−*x*_Se; Se 3d confirmed selenide; Zn 2p gave typical Zn^2+^ from ZIF-8 (Fig. S2).

Colloidal stability: hydrodynamic size and PDI of CSZG remained stable over 72 h in PBS and PBS + 10% FBS (PDI 0.2 to 0.3; Fig. 2I), with no marked change in UV–Vis absorbance at 290 nm (Fig. S1B). GOx release was pH-dependent: 24-h cumulative release reached 79.89% at pH 6.0 vs. 36.57% (pH 6.5) and 29.10% (pH 7.4) (Fig. 2J). pH-responsive degradability: TEM at pH 6.5 showed edge dissolution of CSZG after 24 h (Fig. 2K); UV–Vis confirmed faster degradation at pH 6.5 than at pH 7.4 (Fig. 2L). Batch-to-batch reproducibility: 3 independent CSZG batches gave consistent hydrodynamic diameter, PDI, and ζ-potential (Table S1).

Cu^+^ release under pH 6.0 (neocuproine assay) showed time-dependent output, approaching ~100% cumulative release within 24 h, with a transient plateau between 12 to 20 h (Fig. S3). This confirms bioavailable Cu^+^ supply from CSZG under tumor-like acidity, supporting both Cu-mediated Fenton-like catalysis and Cu-dependent biological effects.

### Evaluation of catalytic properties

**Fig. 3.**
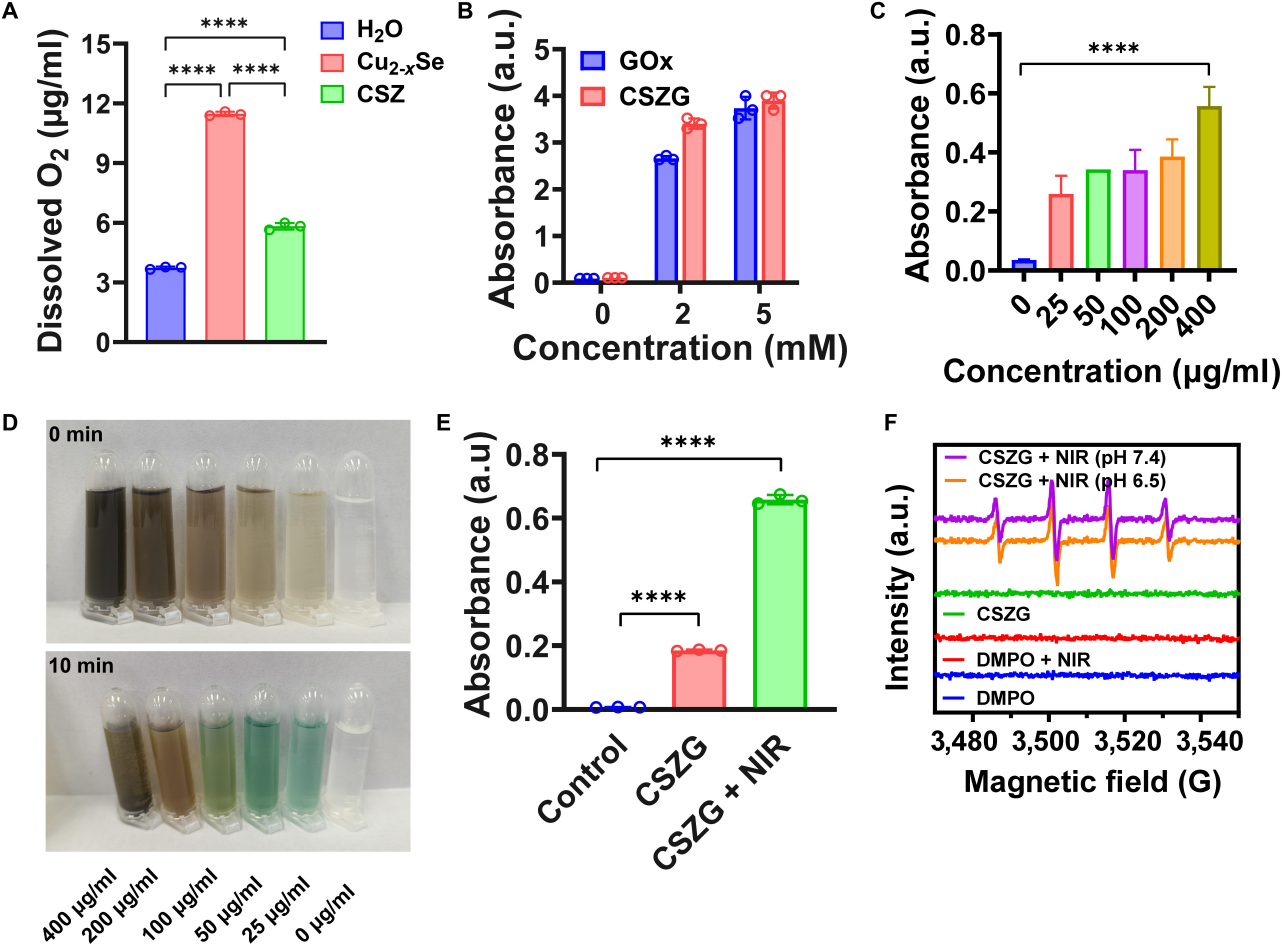
Catalytic properties and •OH generation of CSZG. (A) Dissolved oxygen levels after 5 h of reaction of Cu_2−*x*_Se or CSZ with H_2_O_2_ (125 μM). (B) GOx activity evaluation of free GOx and CSZG using an HRP/ABTS colorimetric assay under different glucose concentrations (0 to 5 mM). (C) UV–Vis absorbance spectra of TMB at 652 nm after reaction with increasing concentrations of CSZG (0 to 400 μg/ml) in the presence of H_2_O_2_. (D) Photographs of the reaction mixtures at 0 and 10 min after TMB addition with increasing concentrations of CSZG in the presence of H_2_O_2_. (E) •OH generation by CSZG with or without 808-nm laser irradiation (1 W/cm^2^, 1 h), assessed using a TMB probe. (F) ESR spectra of DMPO-•OH adducts in different reaction systems, including DMPO, DMPO + NIR, CSZG, CSZG + NIR at pH 7.4, and CSZG + NIR at pH 6.5. Data are presented as mean ± SD (*n* = 3).

Cu_2−*x*_Se generated dissolved O₂ efficiently from H_2_O_2_, whereas CSZ showed weaker activity under the same conditions (Fig. 3A), consistent with partial catalytic-site shielding by the ZIF-8 matrix. GOx activity retention was detected by HRP/ABTS colorimetric assay. As shown in Fig. 3B, both free GOx and CSZG gave glucose-concentration-dependent increases in *A*_415_, confirming that GOx retained glucose-responsive H_2_O_2_-generating activity after encapsulation, thereby supplying H_2_O_2_ for downstream Cu-mediated Fenton-like reactions.

The •OH-generating capacity of CSZG was further evaluated. *A*_652_ increased with CSZG concentration in the presence of H_2_O_2_ (Fig. 3C). The visual color shift from brown (0 min) to blue/blue-green (10 min) indicated progressive •OH-mediated TMB oxidation (Fig. 3D). NIR further enhanced •OH: CSZG + NIR gave higher *A*_652_ than CSZG alone (Fig. 3E).

ESR spectroscopy with DMPO spin trapping was further performed to directly verify •OH generation. DMPO and DMPO + NIR alone gave negligible quartet signals, confirming that NIR alone does not generate detectable •OH. CSZG produced the characteristic DMPO–•OH quartet; the signal was markedly enhanced for CSZG + NIR at pH 7.4 and further intensified at pH 6.5 (Fig. 3F). Thus, CSZG generates •OH in an NIR-enhanced, acidity-amplified manner.

### In vitro biocompatibility, cellular uptake, and apoptosis

**Fig. 4.**
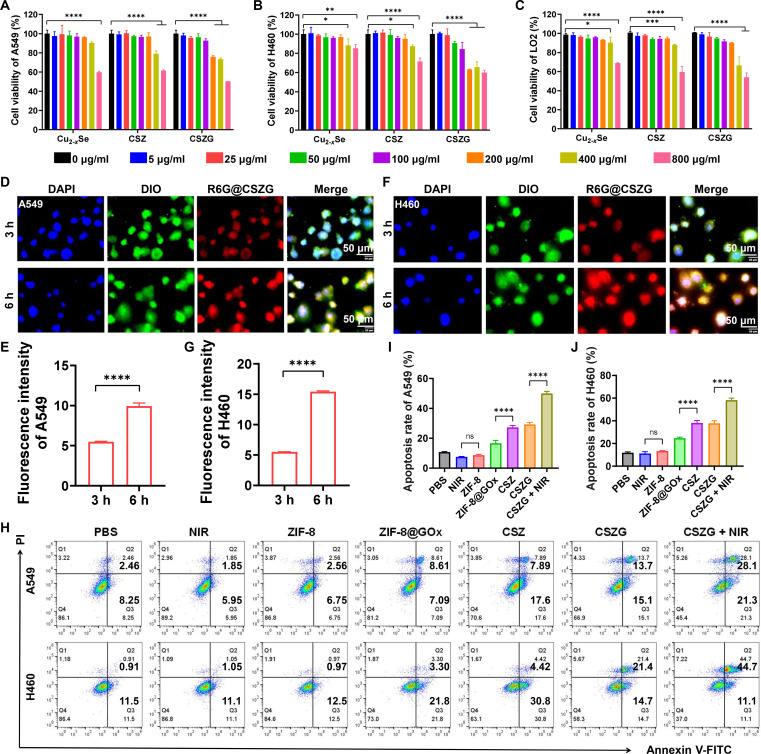
In vitro biosafety, cellular uptake, and apoptosis-inducing activity of CSZG. (A to C) Cell viability of (A) A549, (B) H460, and (C) LO2 cells after 24-h treatment with Cu_2−*x*_Se, CSZ, and CSZG at indicated concentrations. (D and F) Representative CLSM images of (D) A549 and (F) H460 cells incubated with R6G-labeled CSZG (red) for 6 h; nuclei were counterstained with DAPI (blue). Scale bar = 50 μm. (E and G) Quantitative analysis of fluorescence intensity corresponding to (D) and (F). (H) Representative Annexin V-FITC/PI flow cytometry plots. (I and J) Statistical quantification of total apoptotic rates in (I) A549 and (J) H460 cells. Data are presented as mean ± SD (*n* = 3). Statistical significance was determined by one-way ANOVA with Tukey’s post hoc test (*P < 0.05, **P < 0.01, ***P < 0.001, ****P < 0.0001; ns, not significant).

The biocompatibility and anticancer efficacy of CSZG were evaluated in A549 and H460 lung cancer cell lines. CCK-8 assay results showed that Cu_2−*x*_Se, CSZ, and CSZG maintained >90% viability in A549 and H460 at ≤100 μg/ml (Fig. 4A and B). In normal LO2 hepatocytes, viability remained >90% even at 200 μg/ml (Fig. 4C). Hemolysis rate across tested concentrations was <5%, conforming to ISO 10993-4 (Fig. S4).

The cellular internalization of CSZG was visualized using R6G labeling. CLSM showed progressive cytoplasmic red fluorescence increase over 6 h in A549 and H460 (Fig. 4D to G). Apoptosis induction was quantified by flow cytometry (Fig. 4H to J). In A549, apoptotic rates were 10.72% (PBS), 7.8% (NIR), 9.31% (ZIF-8), 15.7% (ZIF-8@GOx), 28.8% (CSZG), and 49.4% (CSZG + NIR). H460 showed a similar trend, with CSZG + NIR reaching 55.8%.

### NIR-enhanced ROS production and cuproptosis-associated mitochondrial alterations in vitro

**Fig. 5.**
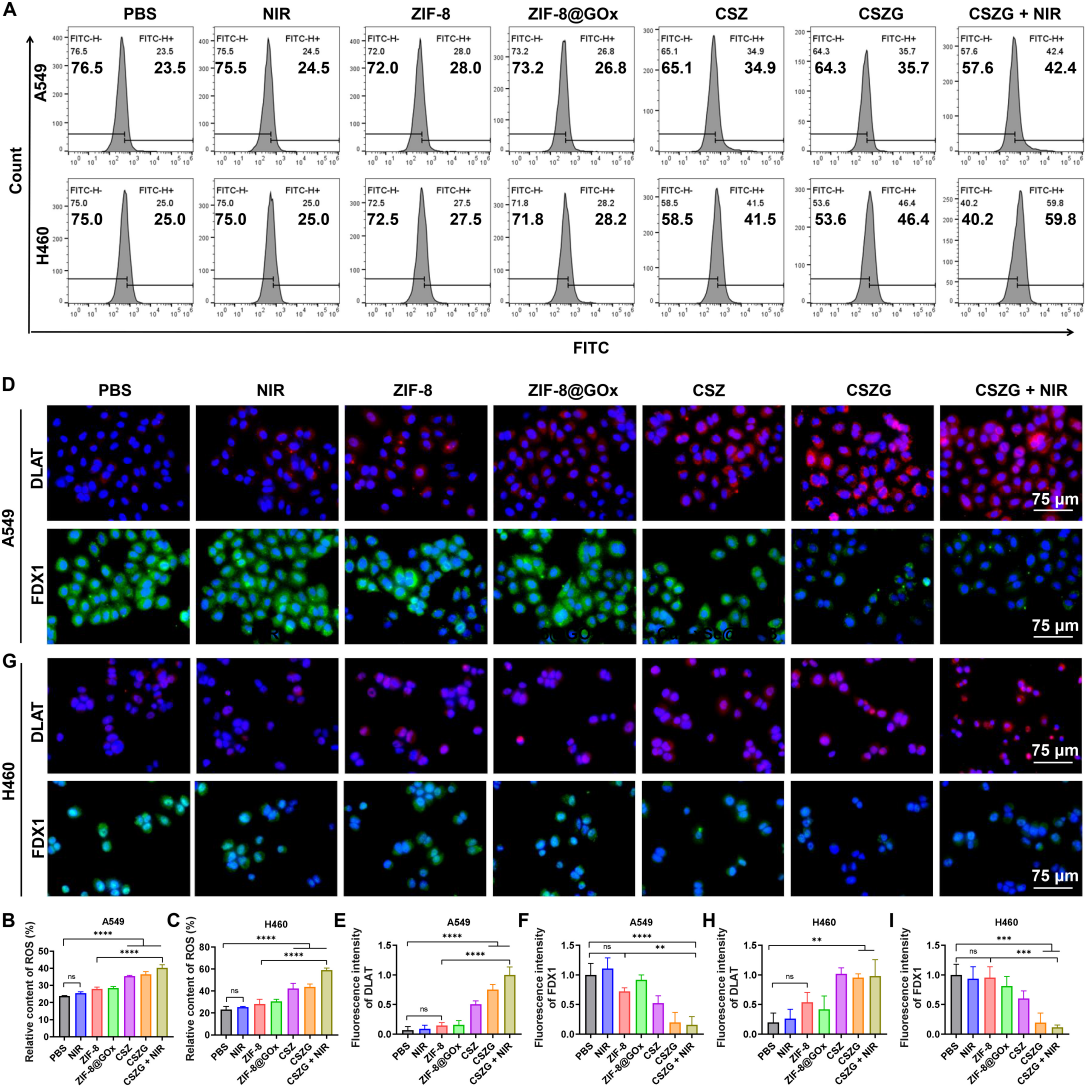
NIR-enhanced ROS generation and cuproptosis-associated mitochondrial alterations in vitro. (A) Representative DCFH-DA flow cytometry plots showing intracellular ROS levels in A549 and H460 cells following indicated treatments. (B and C) Quantitative analysis of the ROS-positive cell populations in (B) A549 and (C) H460 cells. (D) Representative immunofluorescence images of DLAT (red) and FDX1 (green) in A549 cells (scale bar: 75 μm). (E and F) Quantitative analysis of (E) DLAT and (F) FDX1 fluorescence intensities in A549 cells. (G) Representative immunofluorescence images of DLAT and FDX1 in H460 cells (scale bar: 75 μm). (H and I) Quantitative analysis of (H) DLAT and (I) FDX1 fluorescence intensities in H460 cells. Data are presented as mean ± SD (*n* = 3). Statistical significance was determined by one-way ANOVA with Tukey’s post hoc test (*P < 0.05, **P < 0.01, ***P < 0.001, ****P < 0.0001; ns, not significant).

To assess NIR-enhanced oxidative stress, intracellular ROS levels were quantified via DCFH-DA staining and flow cytometry (Fig. 5A). A549 ROS-positive was minimal in PBS/NIR/ZIF-8, moderate in ZIF-8@GOx and CSZG, and 42.4% in CSZG + NIR (Fig. 5B). A similar trend was observed in H460 cells, where the CSZG + NIR group showed the highest ROS level, significantly greater than the PBS control (25.0%) (Fig. 5C).

Cuproptosis-associated mitochondrial alterations were evaluated by monitoring the expression and localization of DLAT and FDX1 using IF staining. A549 CSZG + NIR showed clustered DLAT signals (aggregation) and reduced FDX1 intensity (Fig. 5D to F). H460 CSZG + NIR showed pronounced DLAT clustering and the lowest FDX1 signal among all groups (Fig. 5G to I). These suggest that CSZG + NIR alters the expression/localization of cuproptosis-associated mitochondrial proteins.

**Fig. 6.**
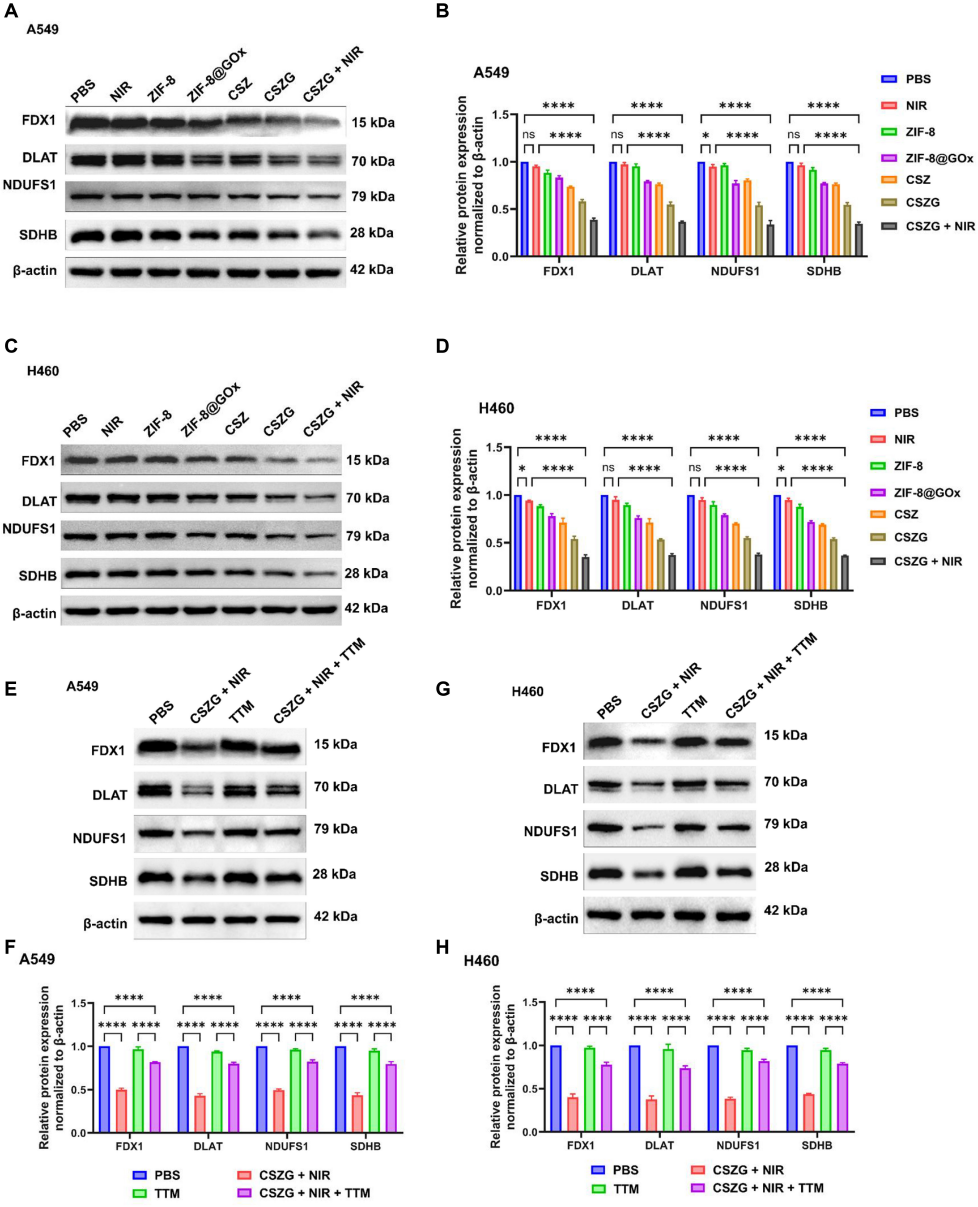
Western blot validation of copper-dependent mitochondrial dysfunction with cuproptosis-associated features and TTM rescue in vitro. (A and B) Representative Western blot images and quantitative analysis of FDX1, DLAT, NDUFS1, and SDHB protein levels in A549 cells after treatment with PBS, NIR, ZIF-8, ZIF-8@GOx, CSZ, CSZG, or CSZG + NIR, respectively. (C and D) Representative Western blot images and quantitative analysis of FDX1, DLAT, NDUFS1, and SDHB protein levels in H460 cells after the same treatments, respectively. (E and F) Representative Western blot images and quantitative analysis of the effects of TTM pretreatment on CSZG + NIR-induced changes in FDX1, DLAT, NDUFS1, and SDHB protein levels in A549 cells, respectively. (G and H) Representative Western blot images and quantitative analysis of the effects of TTM pretreatment in H460 cells, respectively. Protein levels were normalized to β-actin. The corresponding uncropped Western blot images are shown in Figs. S5 to S8. Data are presented as 30 mean ± SD (*n* = 3). Statistical significance was determined by one-way ANOVA with Tukey’s post hoc test (*P < 0.05, **P < 0.01, ***P < 0.001, ****P < 0.0001; ns, not significant).

To further determine whether CSZG + NIR induced cuproptosis-associated mitochondrial dysfunction rather than nonspecific oxidative stress alone, Western blot analysis was performed to examine FDX1, DLAT, and the Fe–S cluster proteins NDUFS1 and SDHB. In both A549 and H460, CSZG + NIR produced the most pronounced decreases across all 4 proteins, whereas PBS/NIR/ZIF-8 showed no obvious change (Fig. 6A to D). Single-module controls (ZIF-8@GOx, CSZ) induced only moderate changes. Together with DLAT aggregation by IF, these data indicate that CSZG + NIR disrupts mitochondrial protein homeostasis and promotes Fe–S loss, consistent with cuproptosis-associated mitochondrial injury (uncropped blots: Figs. S5 and S6).

To verify the copper dependence of these mitochondrial alterations, cells were pretreated with TTM, a copper chelator. TTM alone did not markedly alter FDX1/DLAT/NDUFS1/SDHB. TTM pretreatment partially reversed the CSZG + NIR-induced decreases in both cell lines (Fig. 6E to H; uncropped: Figs. S7 and S8), confirming Cu dependence. Therefore, CSZG + NIR-induced death involves Cu-dependent mitochondrial dysfunction with cuproptosis-associated features, not solely general ROS.

### CSZG promotes M1-like polarization of macrophage-like cells

**Fig. 7.**
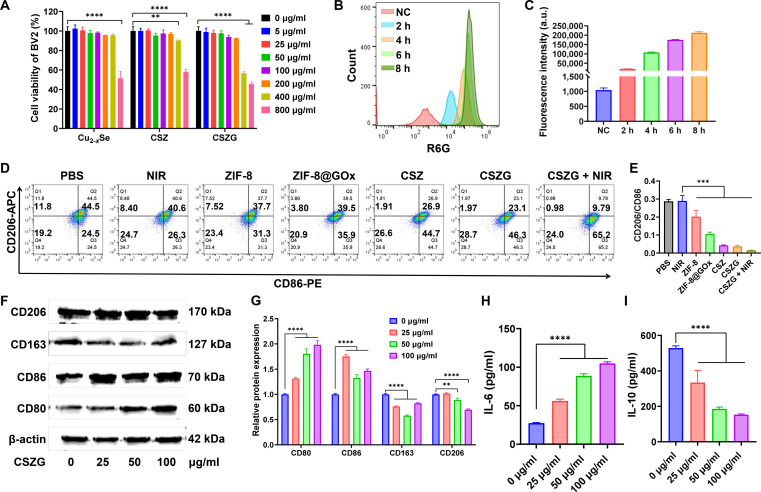
CSZG promotes M1-like polarization of macrophage-like cells in vitro. (A) Cell viability of BV2 cells after 24 h of treatment with Cu_2−*x*_Se, CSZ, or CSZG at the indicated concentrations, assessed by CCK-8 assay. (B) Flow cytometry histograms showing cellular uptake of R6G-labeled CSZG in BV2 cells at 2, 4, 6, and 8 h. (C) Quantitative analysis of the mean fluorescence intensity (MFI) of R6G uptake. (D) Representative flow cytometry plots of IL-4-induced M2-like BV2 cells stained for CD86 and CD206 after different treatments. (E) Quantitative analysis of CD86^+^ and CD206^+^ cell populations and the CD206/CD86 ratio. (F) Western blot analysis of M1-associated markers (CD80 and CD86) and M2-associated markers (CD163 and CD206). (G) Quantitative analysis of protein expression levels normalized to β-actin. (H and I) ELISA analysis of (H) IL-6 and (I) IL-10 levels in culture supernatants. Data are presented as mean ± SD (*n* = 3). Statistical significance was determined by one-way ANOVA with Tukey’s post hoc test (*P < 0.05, **P < 0.01, ***P < 0.001, ****P < 0.0001; ns, not significant).

The immunomodulatory effect of CSZG was evaluated in IL-4-induced M2-like BV2 cells. CCK-8 assays showed negligible cytotoxicity at concentrations up to 200 μg/ml (Fig. 7A). R6G–CSZG uptake was time-dependent by flow and imaging (Fig. 7B and C). To evaluate macrophage-related polarization, CD86 and CD206 expression was analyzed by flow cytometry. CSZG + NIR increased CD86^+^ to 65.2% and reduced CD206^+^ to 0.98%, dropping CD206/CD86 from 0.29 (PBS) to 0.01 (Fig. 7D and E). Western blot analysis further showed that CSZG + NIR up-regulated M1 markers (CD80 and CD86) and down-regulated M2 markers (CD163 and CD206) (Fig. 7F and G). In addition, ELISA analysis showed increased IL-6 secretion and decreased IL-10 secretion in the CSZG + NIR group (Fig. 7H and I). Collectively, CSZG + NIR promotes M1-like and suppresses M2-like polarization in vitro.

### In vivo biosafety and biodistribution

**Fig. 8.**
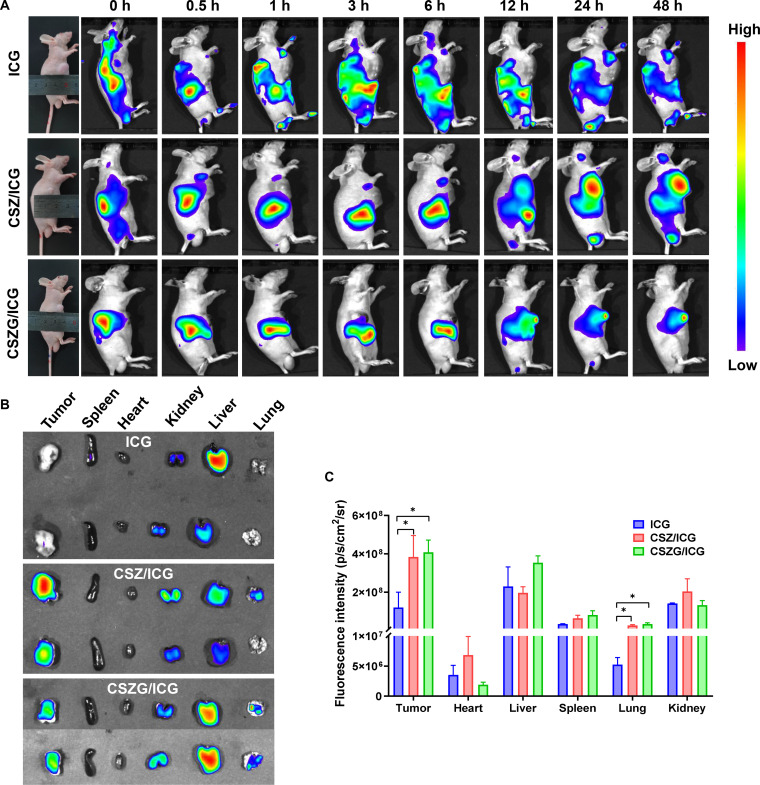
In vivo biodistribution and preliminary biosafety evaluation. (A) In vivo fluorescence images showing the biodistribution of free ICG, CSZ/ICG, and CSZG/ICG at 0, 0.5, 1, 3, 6, 12, 24, and 48 h postinjection. (B) Ex vivo fluorescence images of tumors and major organs (heart, liver, spleen, lung, and kidney) collected at 48 h postinjection. (C) Quantitative fluorescence intensity analysis of tumors and major organs. Data are presented as mean ± SD (*n* = 5). Statistical significance was determined by one-way ANOVA with Tukey’s post hoc test (*P < 0.05, **P < 0.01, ***P < 0.001, ****P < 0.0001; ns, not significant).

To evaluate preliminary systemic biosafety, an acute toxicity study was conducted. Dose-dependent injury was observed only at the highest CSZG dose (20 mg/kg) at 24 h; ZIF-8 alone up to 20 mg/kg showed no marked pathological changes (Figs. S9 and S10 and Table S2). Based on this, 10 mg/kg CSZG was selected for subsequent in vivo studies. The biodistribution of CSZG was characterized using ICG-labeled nanoplatforms. Free ICG cleared rapidly with minimal tumor accumulation. CSZ/ICG and CSZG/ICG showed progressive tumor fluorescence, peaking at 24 h and persisting to 48 h (Fig. 8A). Ex vivo at 48 h confirmed pronounced tumor accumulation of CSZG vs. free ICG (Fig. 8B and C).

### In vivo antitumor efficacy

**Fig. 9.**
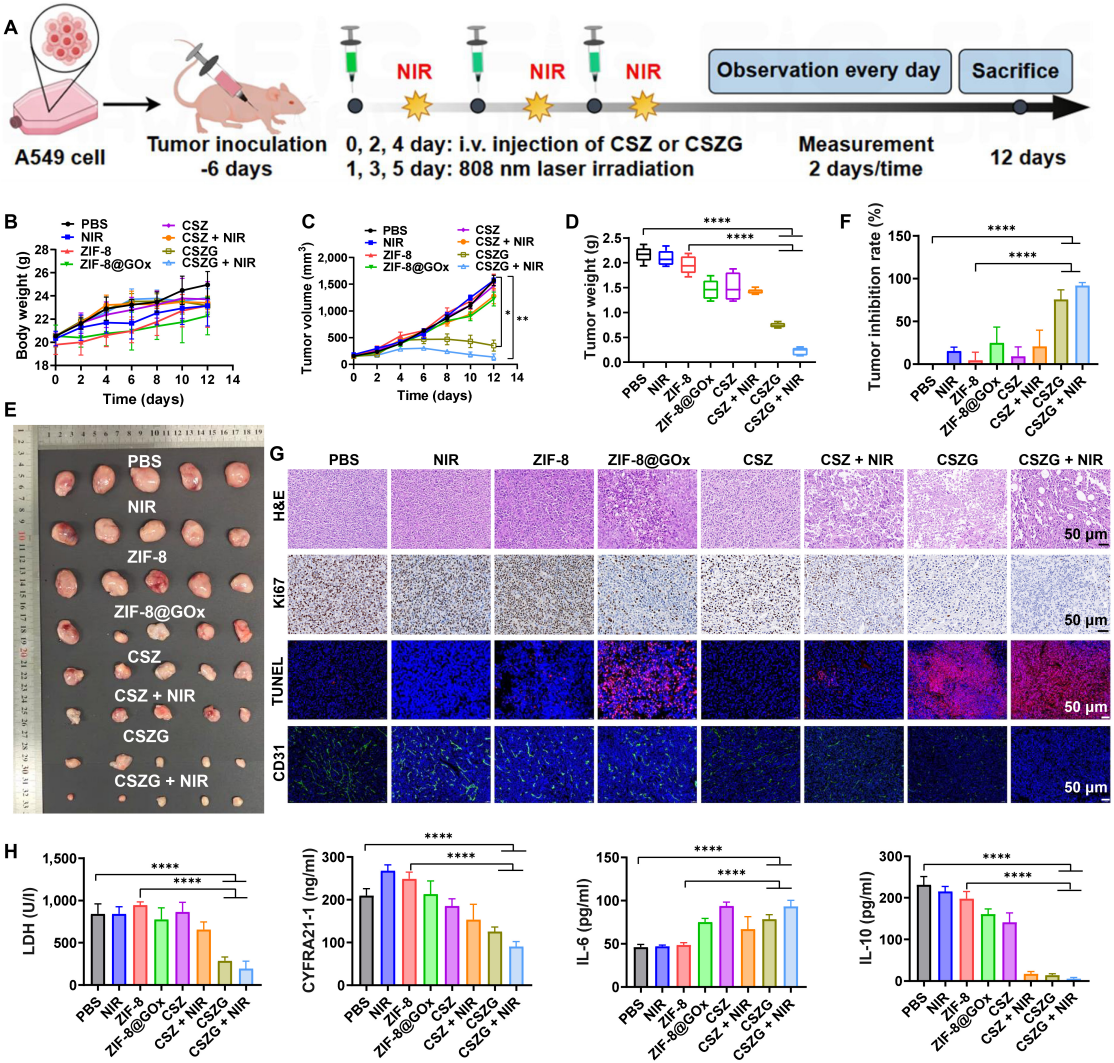
In vivo antitumor efficacy and histological evaluation of nanoplatforms in a subcutaneous lung cancer model. (A) Schematic illustration of the therapeutic protocol and treatment timeline. The treatment groups included PBS, NIR, ZIF-8, ZIF-8@GOx, CSZ, CSZ + NIR, CSZG, and CSZG + NIR to distinguish the contributions of the carrier, GOx loading, Cu_2−*x*_Se incorporation, and NIR irradiation. (B and C) Dynamic changes in (B) body weight and (C) tumor volume during the 12-day treatment period (*n* = 5 mice per group). (D to F) Evaluation of therapeutic outcomes showing (D) final tumor weights, (E) representative photographs of excised tumors, and (F) calculated tumor inhibition rates (*n* = 5 mice per group). (G) Representative histological and immunofluorescence images of tumor sections stained with H&E, Ki67, TUNEL, and CD31. (H) ELISA analysis of serum levels of LDH, CYFRA21-1, IL-6, and IL-10 (*n* = 3). Data are presented as mean ± SD. Statistical significance was determined by one-way ANOVA followed by Tukey’s post hoc test (*P < 0.05, **P < 0.01, ***P < 0.001, ****P < 0.0001; ns, not significant).

The antitumor efficacy of CSZG was systematically evaluated in a subcutaneous A549 lung tumor-bearing mouse model using component-specific formulation controls (Fig. 9A). After 12 days of treatment, tumor volumes in the PBS, NIR-only, and ZIF-8 groups increased rapidly, reaching approximately 10.2-fold of their initial sizes, indicating that NIR irradiation alone or the ZIF-8 carrier alone had negligible antitumor effects (Fig. 9B to F). NIR-only and ZIF-8 had minimal effects, ZIF-8@GOx showed moderate suppression (GOx H_2_O_2_ self-supply), CSZ showed modest activity (Cu_2−*x*_Se catalytic + Cu stress), CSZ + NIR enhanced vs. CSZ alone (NIR amplification), and CSZG + NIR gave the strongest suppression, outperforming CSZG alone (75.6%) and reaching a tumor inhibition rate of 91.7%. Body weight remained stable across groups (Fig. 9B).

Histological analysis further supported the antitumor efficacy of CSZG + NIR. H&E staining showed extensive tumor tissue damage and necrotic areas in the CSZG + NIR group, whereas TUNEL staining revealed increased tumor cell apoptosis. Ki67 and CD31 immunostaining further demonstrated reduced tumor cell proliferation and angiogenesis, respectively (Fig. 9G). Quantitative fluorescence analysis confirmed that CSZG + NIR significantly increased TUNEL-positive apoptotic signals and decreased CD31-positive microvessel signals compared with the other groups (Fig. S11A and B). In addition, ELISA analysis showed reduced serum levels of the tumor-associated markers LDH and CYFRA21-1 in the CSZG and CSZG + NIR groups (Fig. 9H). Flow cytometry analysis of splenocytes from mice receiving PBS, CSZ, CSZ + NIR, CSZG, or CSZG + NIR showed increased proportions of CD4^+^ and CD8^+^ cells in the CSZG and CSZG + NIR groups, suggesting treatment-associated changes in systemic immune cell populations (Fig. S12).

### In vivo cuproptosis-associated mitochondrial alterations and TAM remodeling

**Fig. 10.**
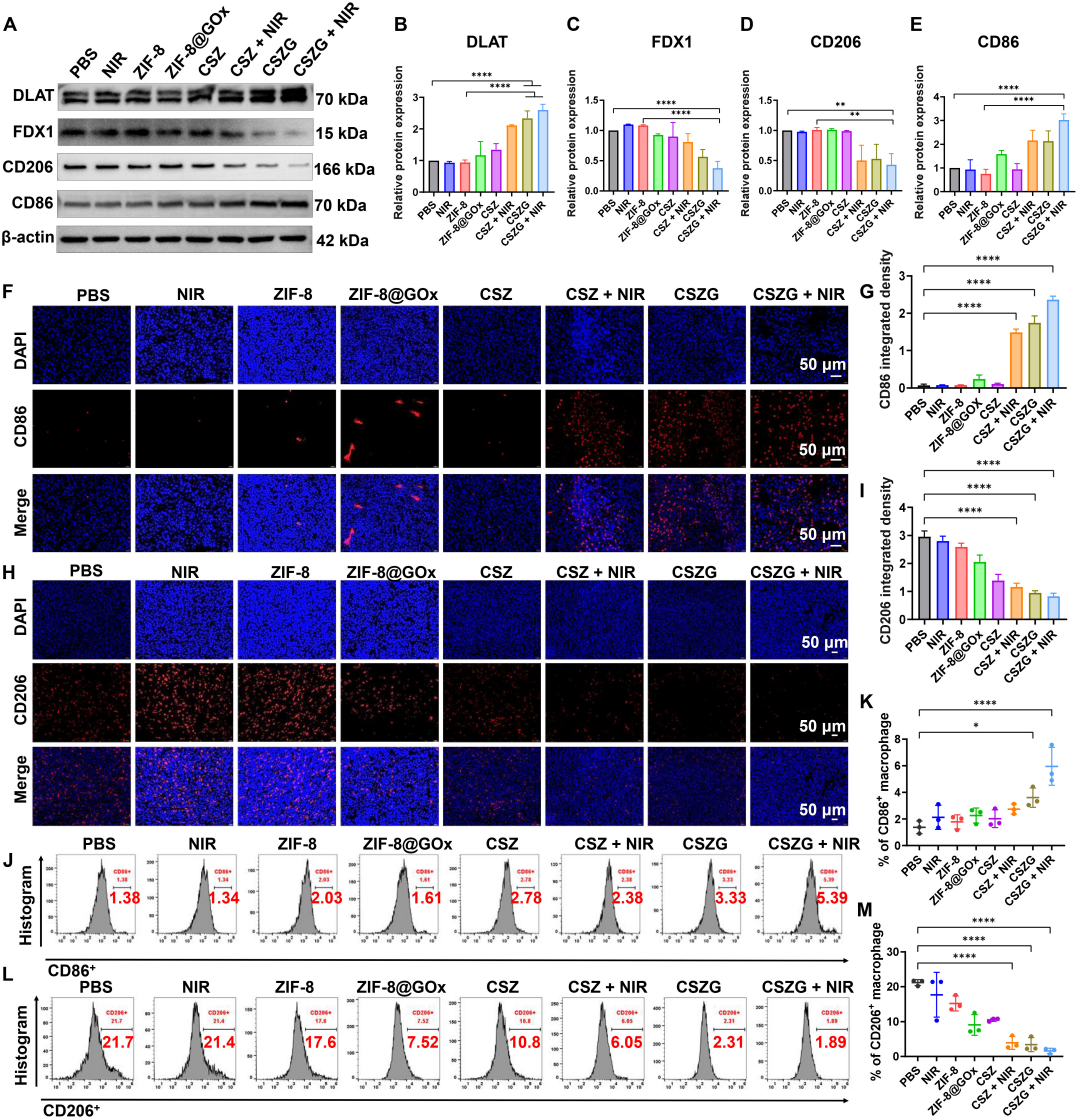
In vivo cuproptosis-associated mitochondrial alterations and macrophage-related immune remodeling. (A) Western blot analysis of DLAT, FDX1, CD206, and CD86 in tumor tissues. (B and C) Quantification of (B) DLAT and (C) FDX1 expression levels normalized to β-actin (*n* = 3). (D and E) Quantification of (D) CD206 and (E) CD86 expression levels normalized to β-actin (*n* = 3). (F and G) Representative immunofluorescence images and quantitative fluorescence analysis of CD86-positive signals in tumor sections, respectively (*n* = 3). Scale bar: 50 μm. (H and I) Representative immunofluorescence images and quantitative fluorescence analysis of CD206-positive signals in tumor sections, respectively (*n* = 3). Scale bar: 50 μm. (J and K) Representative flow cytometry histograms and quantitative analysis of CD86^+^ cells within the CD11b^+^ tumor-infiltrating myeloid/macrophage-enriched population, respectively (*n* = 3). (L and M) Representative flow cytometry histograms and quantitative analysis of CD206^+^ cells within the CD11b^+^ tumor-infiltrating myeloid/macrophage-enriched population, respectively (*n* = 3). Data are presented as mean ± SD. Statistical significance was determined by one-way ANOVA with Tukey’s post hoc test (*P < 0.05, **P < 0.01, ***P < 0.001, ****P < 0.0001; ns, not significant).

To further examine whether cuproptosis-associated mitochondrial alterations also occurred in vivo, the expression of DLAT and FDX1 in tumor tissues was analyzed (Fig. 10A). No obvious changes in FDX1 or DLAT expression were observed in the PBS, NIR-only, or ZIF-8 groups. In contrast, tumors from the CSZG and CSZG + NIR groups showed DLAT aggregation and reduced FDX1 expression, with more pronounced changes in the CSZG + NIR group (Fig. 10B and C). These in vivo findings were consistent with the cellular results and support the contribution of cuproptosis-associated mitochondrial dysfunction to the antitumor activity of CSZG + NIR.

Macrophage-related immune polarization in tumor tissues was evaluated by Western blotting, IF staining, and flow cytometry. Western blot analysis showed that CSZG and CSZG + NIR increased the expression of the M1-associated marker CD86 and decreased the expression of the M2-associated marker CD206, with the most pronounced changes observed in the CSZG + NIR group (Fig. 10D and E). Consistently, IF staining showed increased CD86-positive signals and reduced CD206-positive signals in tumor sections after CSZG + NIR treatment (Fig. 10F and H). Quantitative fluorescence analysis further confirmed that CSZG + NIR significantly increased CD86-positive signals and decreased CD206-positive signals compared with the control groups (Fig. 10G and I).

To further validate immune polarization in the tumor microenvironment, flow cytometric analysis was performed using tumor tissues collected from the 8 treatment groups. The gating strategy is shown in Fig. S13. Within the CD11b^+^ tumor-infiltrating myeloid/macrophage-enriched population, CSZG + NIR markedly increased the proportion of CD86^+^ cells and decreased the proportion of CD206^+^ cells compared with the PBS, NIR, and ZIF-8 groups (Fig. 10J to M). These results, together with the in vitro flow cytometry, Western blotting, cytokine, and IF data, provide comprehensive evidence that CSZG + NIR promotes M1-like immune polarization and suppresses M2-like polarization in the tumor microenvironment.

At the end of treatment, systemic biosafety was further evaluated. Serum biochemical analysis showed no significant abnormalities in ALT, AST, UREA, or CREA levels among the treatment groups, indicating no apparent hepatic or renal toxicity under the tested treatment conditions (Fig. S14). In addition, H&E staining of major organs from mice receiving PBS, CSZ, CSZ + NIR, CSZG, or CSZG + NIR revealed no obvious pathological abnormalities (Fig. S15), and the hemolysis rate remained below 5% (Fig. S16). Together with body weight monitoring, these results support the favorable short-term systemic biosafety and hemocompatibility of CSZG under the tested therapeutic conditions.

## Discussion

In this study, the antitumor performance of CSZG arises from functional coupling rather than simple coloading. Component-specific controls support this design logic: NIR-only and ZIF-8 alone were insufficient; ZIF-8@GOx showed moderate efficacy (GOx H_2_O_2_ self-supply); CSZ showed Cu_2−*x*_Se-mediated catalytic + Cu stress; CSZ + NIR > CSZ (NIR amplification); CSZG + NIR > all partial combinations (GOx + Cu_2−*x*_Se + NIR + ZIF-8 pH delivery). Free GOx and bare Cu_2−*x*_Se were not used as primary mechanistic controls because they differ substantially from the formulated nanoplatform in stability, uptake, biodistribution, and in vivo exposure; hence, ZIF-8@GOx and CSZ served as formulation-matched module controls.

Metal-containing and inorganic nanomaterials are increasingly exploited in biomedical catalysis because their composition-dependent redox, surface chemistry, and catalytic behavior can be rationally tuned for therapy [16–18]. In CSZG, ZIF-8 provides an acid-labile carrier, GOx supplies glucose-responsive H_2_O_2_, Cu_2−*x*_Se delivers Cu^+^ for Fenton-like conversion and Cu-dependent mitochondrial stress, and NIR irradiation amplifies ROS—likely assisted by the documented photothermal property of Cu_2−*x*_Se—so that each component reinforces the others in a cascade addressing selectivity, catalytic efficiency, and single-pathway resistance [32–36].

Physicochemically, the loss of crystalline ZIF-8 peaks after Cu_2−*x*_Se incorporation suggests substantial framework reorganization. Rather than mere collapse, this reduced crystallinity likely reflects successful guest incorporation and may favor acid-triggered disassembly, as reported for enzyme@ZIF-8 composites [37,38]. Accelerated GOx release at acidic pH aligns with this and benefits tumor-specific activation [39]. The balance between physiological stability and acid-enhanced degradation represents a workable trade-off for systemic robustness vs. intratumoral responsiveness.

Catalytically, CSZ showed weaker H_2_O_2_ decomposition than free Cu_2−*x*_Se, plausibly from catalytic-site shielding by ZIF-8. This trade-off is acceptable given improved stability and selective tumor activation. XPS confirmed low-valence Cu, selenide, and Zn^2+^ from ZIF-8, supporting composite formation. Cu^+^ release under pH 6.0 confirmed bioavailable Cu^+^ output for Fenton-like and Cu-dependent biology. HRP/ABTS confirmed retained GOx activity post-encapsulation, critical for H_2_O_2_ self-supply [40,41]. ESR directly verified •OH generation and NIR/pH amplification, consistent with prior NIR-enhanced Fenton reports [42].

The Western blot + TTM rescue data further support Cu-dependent mitochondrial dysfunction in CSZG + NIR-induced death. FDX1/DLAT reduction + Fe–S (NDUFS1, SDHB) depletion indicate mitochondrial protein homeostasis disruption consistent with cuproptosis-associated injury [43]; FDX1 also regulates protein lipoylation via lipoic acid synthetase [44], linking back to cuproptosis. TTM partially rescued these alterations, confirming Cu dependence. DLAT aggregation by IF adds evidence beyond general oxidative stress. Nevertheless, because ROS generation, GOx-mediated glucose oxidation, and Cu-mediated catalytic reactions occur concurrently, cuproptosis-associated mitochondrial dysfunction should be interpreted as an important contributing mechanism rather than the sole mode of tumor cell death.

The immune-related findings extend the significance of CSZG beyond direct tumor killing. In vitro, CSZG + NIR increased CD86 expression, reduced CD206 expression, up-regulated CD80/CD86, down-regulated CD163/CD206, and shifted cytokine secretion toward increased IL-6 and decreased IL-10. In vivo, IF staining and flow cytometry further showed increased CD86-positive and decreased CD206-positive populations in tumor tissues, particularly within CD11b^+^ tumor-infiltrating myeloid/macrophage-enriched cells. These results provide more comprehensive evidence that CSZG + NIR remodels the tumor immune microenvironment toward an M1-like phenotype. Previous studies have also shown that Cu_2−*x*_Se-based nanomaterials can mediate ROS-related macrophage repolarization and enhance antitumor immune responses [45]. Therefore, the therapeutic effect of CSZG + NIR likely reflects both direct tumoricidal stress and macrophage-related immune microenvironment remodeling.

Compared with recently reported multifunctional lung cancer nanoplatforms, CSZG emphasizes a cascade-amplified therapeutic logic rather than simple functional combination. Previously reported nanoplatforms have achieved improved lung cancer therapy through photothermal/CDT, photodynamic therapy, targeting delivery, or imaging-guided treatment [46–49]. In contrast, the present CSZG system integrates GOx-mediated glucose oxidation and H₂O₂ self-supply, Cu_2−*x*_Se-mediated catalytic reactions and copper-dependent mitochondrial dysfunction, ZIF-8-based pH-responsive delivery, and NIR-enhanced therapeutic activation within one biodegradable platform. This design enables multiple therapeutic modules to reinforce each other at the tumor site, which may explain the stronger tumor inhibition observed in the CSZG + NIR group compared with the single-module and partial-combination controls.

In vivo, CSZG + NIR achieved 91.7% TIR with favorable short-term systemic biosafety under tested conditions (BW, serum biochemistry, H&E, and hemolysis). Nanoparticle dose, surface coating, and surface density can modulate circulation, immune clearance, and tissue bioreaction [29–31]; hence, systematic biosafety evaluation remains essential for nanomedicine development. Long-term biosafety was not fully assessed here; extended pharmacokinetics, clearance, immunotoxicity, and chronic toxicity will be required before translational consideration.

Several limitations should be considered. (a) Direct photothermal characterization (temperature curves and conversion efficiency) was not performed; NIR enhancement was inferred from ROS data and Cu_2−*x*_Se literature [42]. (b) Quantitative Cu_2−*x*_Se loading in CSZG was not determined; instead, batch Cu^+^ release was characterized (Fig. S3). (c) BET was omitted; given the XRD-confirmed amorphous/disordered structure, BET may be of limited predictive value here. (d) Macrophage remodeling was shown, but broader immune profiling would further clarify the immunomodulatory effects of CSZG in future studies.

## Conclusion

In this study, we developed a biodegradable ZIF-8-based nanosystem, CSZG, coloading GOx and Cu-rich Cu_2−*x*_Se for multimodal lung cancer therapy. CSZG exhibits pH-responsive degradability, sustained GOx release under acidity, retained glucose-responsive GOx activity, favorable colloidal stability, and reproducible preparation. Mechanistically, CSZG enables GOx-mediated H_2_O_2_ self-supply, NIR-enhanced •OH generation, Cu-dependent mitochondrial dysfunction with cuproptosis-associated features, and macrophage repolarization toward an M1-like phenotype. In an A549 xenograft model, CSZG + NIR achieved a 91.7% tumor inhibition rate with favorable short-term systemic biosafety under tested conditions. This work positions CSZG as a promising multimodal nanoplatform integrating CDT, Cu-dependent mitochondrial stress, and immune remodeling for lung cancer. Future studies should evaluate orthotopic/metastatic models and long-term biosafety/systemic immune responses.

## Ethical Approval

This study has been approved by the ethics committee of the Zhongshan Hospital Affiliated to Fudan University (Shanghai, China) and conducted in accordance with ethical guidelines.

## Data Availability

The data will be made available on request.

## References

[1] Bray F, Laversanne M, Sung H, Ferlay J, Siegel RL, Soerjomataram I, Jemal A. Global cancer statistics 2022: GLOBOCAN estimates of incidence and mortality worldwide for 36 cancers in 185 countries. CA Cancer J Clin. 2024;74(3):229–263. 38572751 10.3322/caac.21834

[2] Leiter A, Veluswamy RR, Wisnivesky JP. The global burden of lung cancer: Current status and future trends. Nat Rev Clin Oncol. 2023;20(9):624–639. 37479810 10.1038/s41571-023-00798-3

[3] Gridelli C, Rossi A, Carbone DP, Guarize J, Karachaliou N, Mok T, Petrella F, Spaggiari L, Rosell R. Non-small-cell lung cancer. Nat Rev Dis Primers. 2015;1:15009. 27188576 10.1038/nrdp.2015.9

[4] Saleem HM, Ramaiah P, Gupta J, Jalil AT, Kadhim NA, Alsaikhan F, Ramírez-Coronel AA, Tayyib NA, Guo Q. Nanotechnology-empowered lung cancer therapy: From EMT role in cancer metastasis to application of nanoengineered structures for modulating growth and metastasis. Environ Res. 2023;232: Article 115942. 37080268 10.1016/j.envres.2023.115942

[5] Zheng X, Song X, Zhu G, Pan D, Li H, Hu J, Xiao K, Gong Q, Gu Z, Luo K, et al. Nanomedicine combats drug resistance in lung cancer. Adv Mater. 2024;36(3): Article e2308977. 37968865 10.1002/adma.202308977

[6] Xu M, Xu Y, Du C, Gu G, Wei G. Biomimetic CuCoO_2_ nanosheets reinforced with self-assembling peptide nanofibers for tumor photothermal therapy. RSC Adv. 2024;14(53):39163–39172. 39664248 10.1039/d4ra07435a PMC11632949

[7] Kah G, Chandran R, Abrahamse H. Green silver nanoparticles curcumin conjugate induced photodynamic therapy of lung cancer and lung cancer stem cells. RSC Adv. 2025;15(7):5020–5041. 39957816 10.1039/d4ra06035k PMC11827557

[8] Jiang C, Zhao Z, East AK, Bandyopadhyay S, Jiang Z, Chan J. Logic-gated approach for targeted delivery and site-selective activation of photothermal agents in precision cancer treatment. Chem Sci. 2025;16(12):5155–5165. 39981039 10.1039/d4sc08228a PMC11837750

[9] Zhao X, Gu T-Y, Xia Y-P, Gao X-M, Chen L-J, Yan L-X, Yan X-P. Self-evolving persistent luminescence nanoprobes for autofluorescence-free ratiometric imaging and on-demand enhanced chemodynamic therapy of pulmonary metastatic tumors. Biomater Sci. 2024;12(12):3229–3237. 38764365 10.1039/d4bm00379a

[10] Du H, Xu E, Xu Y, Xue Q, Xu H, Song J. 3D DNAzyme motor nanodevice with self-powered FRET amplifier and self-supplied H_2_O_2_ for enhancing human neutrophil elastase profiling and chemodynamic therapy in lung tumor. Adv Sci. 2024;11(44): Article e2406599. 10.1002/advs.202406599 PMC11600284 39348241

[11] Rai A, Patwardhan RS, Jayakumar S, Pachpatil P, Das D, Panigrahi GC, Gota V, Patwardhan S, Sandur SK. Clobetasol propionate, a Nrf-2 inhibitor, sensitizes human lung cancer cells to radiation-induced killing via mitochondrial ROS-dependent ferroptosis. Acta Pharmacol Sin. 2024;45(7):1506–1519. 38480835 10.1038/s41401-024-01233-8 PMC11192725

[12] Zheng H, Chen H, Cai Y, Shen M, Li X, Han Y, Deng X, Cao H, Liu J, Li H, et al. Hydrogen sulfide-mediated persulfidation regulates homocysteine metabolism and enhances ferroptosis in non-small cell lung cancer. Mol Cell. 2024;84(20):4016–4030.e6. 39321805 10.1016/j.molcel.2024.08.035

[13] Li K, Wu L, Wang H, Fu Z, Gao J, Liu X, Fan Y, Qin X, Ni D, Wang J, et al. Apoptosis and cuproptosis co-activated copper-based metal-organic frameworks for cancer therapy. J Nanobiotechnology. 2024;22(1):546. 39237931 10.1186/s12951-024-02828-3 PMC11378619

[14] Jawed R, Bhatti H. Cuproptosis in lung cancer: Therapeutic options and prognostic models. Apoptosis. 2024;29(9–10):1393–1398. 38735011 10.1007/s10495-024-01978-x

[15] Abu-Dief AM, Alsehli M, Al-Enizi A, Nafady A. Recent advances in mesoporous silica nanoparticles for targeted drug delivery applications. Curr Drug Deliv. 2022;19(4):436–450. 34238185 10.2174/1567201818666210708123007

[16] Abu-Dief AM, Salaheldeen M, El-Dabea T. Recent advances in development of gold nanoparticles for drug delivery systems. J Mod Nanotechnol. 2021;1(1).

[17] Marzouk AA, Abu-Dief AM, Abdelhamid AA. Hydrothermal preparation and characterization of ZnFe_2_O_4_ magnetic nanoparticles as an efficient heterogeneous catalyst for the synthesis of multi-substituted imidazoles and study of their anti-inflammatory activity. Appl Organomet Chem. 2018;32(1): Article e3794.

[18] Saddik MS, Al-Hakkani MF, Abu-Dief AM, Mohamed MS, Al-Fattah IA, Makki M, El-Mokhtar MA, Sabet MA, Amin MS, Ahmed HA, et al. Formulation and evaluation of azithromycin-loaded silver nanoparticles for the treatment of infected wounds. Int J Pharm X. 2024;7: Article 100245. 38633410 10.1016/j.ijpx.2024.100245 PMC11021372

[19] Abu-Dief AM, Alsehli M. DNA interaction of drug molecules for cancer treatment. J Pharm Nurs. 2020;2:12–16.

[20] Khalil KD, Bashal AH, Habeeb T, Kebeish R, Abu-Dief AM. Multifunctional lanthanum oxide-doped carboxymethyl cellulose nanocomposites: A promising approach for antimicrobial and targeted anticancer applications. Int J Biol Macromol. 2024;283(Pt 1): Article 137495. 39528180 10.1016/j.ijbiomac.2024.137495

[21] Saddik MS, Elsayed MMA, Abdelkader MSA, El-Mokhtar MA, Abdel-Aleem JA, Abu-Dief AM, Al-Hakkani MF, Farghaly HS, Abou-Taleb HA. Novel green biosynthesis of 5-fluorouracil chromium nanoparticles using *Harpullia pendula* extract for treatment of colorectal cancer. Pharmaceutics. 2021;13(2):226. 33562032 10.3390/pharmaceutics13020226 PMC7915530

[22] Wang M, Yu F, Zhang Y. Present and future of cancer nano-immunotherapy: Opportunities, obstacles and challenges. Mol Cancer. 2025;24(2):26. 39827147 10.1186/s12943-024-02214-5 PMC11748575

[23] Chen Y-T, Luo Y-X, Chan S-H, Chiu W-Y, Yang H-W. Dual antibody-aided mesoporous nanoreactor for H_2_O_2_ self-supplying chemodynamic therapy and checkpoint blockade immunotherapy in triple-negative breast cancer. J Nanobiotechnology. 2023;21(1):385. 37875918 10.1186/s12951-023-02154-0 PMC10594761

[24] Zhang X, He C, He X, Fan S, Ding B, Lu Y, Xiang G. HIF-1 inhibitor-based one-stone-two-birds strategy for enhanced cancer chemodynamic-immunotherapy. J Control Release. 2023;356:649–662. 36933701 10.1016/j.jconrel.2023.03.026

[25] Luo D, Carter KA, Razi A, Geng J, Shao S, Giraldo D, Sunar U, Ortega J, Lovell JF. Doxorubicin encapsulated in stealth liposomes conferred with light-triggered drug release. Biomaterials. 2016;75:193–202. 26513413 10.1016/j.biomaterials.2015.10.027 PMC4644481

[26] Zhang L, Jiang C, Li B, Liu Z, Gu B, He S, Li P, Sun Y, Song S. A core-shell Au@Cu_2-x_Se heterogeneous metal nanocomposite for photoacoustic and computed tomography dual-imaging-guided photothermal boosted chemodynamic therapy. J Nanobiotechnology. 2021;19(1):410. 34876141 10.1186/s12951-021-01159-x PMC8650506

[27] Zhang S, Sun C, Zeng J, Sun Q, Wang G, Wang Y, Wu Y, Dou S, Gao M, Li Z. Ambient aqueous synthesis of ultrasmall PEGylated Cu_2-x_Se nanoparticles as a multifunctional theranostic agent for multimodal imaging guided photothermal therapy of cancer. Adv Mater. 2016;28(40):8927–8936. 27560922 10.1002/adma.201602193

[28] Wu C, Tan J, Wang X, Qin C, Long W, Pan Y, Li Y, Liu Q. Pan-cancer analyses reveal molecular and clinical characteristics of cuproptosis regulators. iMeta. 2023;2(1): Article e68. 38868340 10.1002/imt2.68 PMC10989956

[29] Mohamed DS, Abdelhaleem NG, Shaaban EA, Abu-Dief AM. The possible role of nanocurcumin in rat model of statin-induced myopathy: Histological and immunohistochemical study. J Adv Med Med Res. 2022;34:76–88.

[30] Abu-Dief AM, Alsehli M, Awaad A. A higher dose of PEGylated gold nanoparticles reduces the accelerated blood clearance phenomenon effect and induces spleen B lymphocytes in albino mice. Histochem Cell Biol. 2022;157(6):641–656. 35157114 10.1007/s00418-022-02086-0

[31] Abu-Dief AM, Alsehli M, Awaad A. The bioreaction and immune responses of PEG-coated silica NPs and the role of the surface density coating after oral administration into mice. Appl Nanosci. 2023;13(8):5563–5578.

[32] Wang Y, Tang Y, Guo L, Yang X, Wu S, Yue Y, Xu C. Recent advances in zeolitic imidazolate frameworks as drug delivery systems for cancer therapy. Asian J Pharm Sci. 2025;20(1): Article 101017. 39931355 10.1016/j.ajps.2025.101017 PMC11808527

[33] Gao W, Han X, Li L, Xu Y, Xu M, Gao Z, Wang C. Functionalized ZIF-8 as a versatile platform for drug delivery and cancer therapy: Strategies, challenges and prospects. J Mater Chem B. 2025;13(12):3758–3785. 40019146 10.1039/d4tb02289k

[34] Fu L-H, Qi C, Lin J, Huang P. Catalytic chemistry of glucose oxidase in cancer diagnosis and treatment. Chem Soc Rev. 2018;47(17):6454–6472. 30024579 10.1039/c7cs00891k

[35] Jia C, Guo Y, Wu F-G. Chemodynamic therapy via Fenton and Fenton-like nanomaterials: Strategies and recent advances. Small. 2022;18(6):2103868. 10.1002/smll.202103868 34729913

[36] Wu G-L, Tan X, Yang Q. Recent advances on NIR-II light-enhanced chemodynamic therapy. Adv Healthc Mater. 2024;13(10): Article e2303451. 37983596 10.1002/adhm.202303451

[37] Kinoshita M, Yanagida S, Gessei T, Monkawa A. Precursor concentration effects on crystallite size and enzyme immobilization efficiency of enzyme@ZIF-8 composite. J Cryst Growth. 2022;600: Article 126877.

[38] Liang W, Ricco R, Maddigan NK, Dickinson RP, Xu H, Li Q, Sumby CJ, Bell SG, Falcaro P, Doonan CJ. Control of structure topology and spatial distribution of biomacromolecules in protein@ZIF-8 biocomposites. Chem Mater. 2018;30(3):1069–1077.

[39] Feng L, Dong Z, Tao D, Zhang Y, Liu Z. The acidic tumor microenvironment: A target for smart cancer nano-theranostics. Natl Sci Rev. 2018;5(2):269–286.

[40] Gkaniatsou E, Sicard C, Ricoux R, Mahy J-P, Steunou N, Serre C. Metal-organic frameworks: A novel host platform for enzymatic catalysis and detection. Mater Horiz. 2017;4(1):55–63.

[41] Bai J, Peng C, Guo L, Zhou M. Metal-organic framework-integrated enzymes as bioreactor for enhanced therapy against solid tumor via a cascade catalytic reaction. ACS Biomater Sci Eng. 2019;5(11):6207–6215. 33405528 10.1021/acsbiomaterials.9b01200

[42] Luan X, Pan Y, Zhou Y, Zhou D, Zhao W, Zeng F, Zhu Z, Lu Q, Lu Q, Gao Y, et al. Targeted self-assembly of renal clearable Cu_2−x_Se to induce lysosome swelling for multimodal imaging guided photothermal/chemodynamic synergistic therapy. Adv Funct Mater. 2022;32(51):2208354.

[43] Tsvetkov P, Coy S, Petrova B, Dreishpoon M, Verma A, Abdusamad M, Rossen J, Joesch-Cohen L, Humeidi R, Spangler RD, et al. Copper induces cell death by targeting lipoylated TCA cycle proteins. Science. 2022;375(6586):1254–1261. 35298263 10.1126/science.abf0529 PMC9273333

[44] Dreishpoon MB, Bick NR, Petrova B, Warui DM, Cameron A, Booker SJ, Kanarek N, Golub TR, Tsvetkov P. FDX1 regulates cellular protein lipoylation through direct binding to LIAS. J Biol Chem. 2023;299(9): Article 105046. 37453661 10.1016/j.jbc.2023.105046 PMC10462841

[45] Zheng Y, Han Y, Wang T, Liu H, Sun Q, Hu S, Chen J, Li Z. Reprogramming tumor-associated macrophages via ROS-mediated novel mechanism of ultra-small Cu_2-x_Se nanoparticles to enhance anti-tumor immunity. Adv Funct Mater. 2022;32(12):2108971.

[46] Fang D, Jin H, Huang X, Shi Y, Liu Z, Ben S. PPy@Fe_3_O_4_ nanoparticles inhibit tumor growth and metastasis through chemodynamic and photothermal therapy in non-small cell lung cancer. Front Chem. 2021;9: Article 789934. 34820358 10.3389/fchem.2021.789934 PMC8606671

[47] Fang D, Liu Z, Jin H, Huang X, Shi Y, Ben S. Manganese-based prussian blue nanocatalysts suppress non-small cell lung cancer growth and metastasis via photothermal and chemodynamic therapy. Front Bioeng Biotechnol. 2022;10: Article 939158. 35814022 10.3389/fbioe.2022.939158 PMC9257087

[48] Sun H, Zhang L, Zhao N, Xin H. Cu^2+^–citrate–chitosan complex nanoparticles for the chemodynamic therapy of lung cancer. ACS Omega. 2024;9(7):8425–8433. 38405439 10.1021/acsomega.3c09619 PMC10883013

[49] Yin M, Liu L, Yan Y, Wang H, Li W, Dong Y, Kong G. A targeting nanoplatform for chemo-photothermal synergistic therapy of small-cell lung cancer. Int J Cancer. 2024;155(11):2094–2106. 38985144 10.1002/ijc.35065

