# Supplementary material for: A Degradable Nanoplatform Integrating Glucose Oxidase and Copper Selenide for Near-Infrared-Enhanced Multimodal Synergistic Therapy in Lung Cancer
Source: Biomater Res. 2026 Aug 14;30:0404. doi: 10.34133/bmr.0404 (PMC13473715; doi:10.34133/bmr.0404)
Supplement: Supplementary file 1 [file bmr.0404.f1.docx]

**Supplementary Materials**

**A degradable nanoplatform integrating glucose oxidase and copper selenide for near-infrared-enhanced multimodal synergistic therapy in lung cancer**

Yong Zhang^1, ‡, *^, Lei Xue^2, ‡^, Lin Shi^1, ‡^, Mengni Zhu^3^, Jun Li^2^, Degang Liu^2^, Qiang Yang^2^, Jiebai Zhou^1^, Zilong Liu^1^, Dawei Yang^1^, Jiangzhou Peng^2, *^

1. Department of Pulmonary, Zhongshan Hospital, Fudan University, Shanghai, 200032, China

2. Department of Thoracic Surgery, The Third Affiliated Hospital of Southern Medical University, Guangzhou, 510630, China

3. Shanghai Key Laboratory of Magnetic Resonance, School of Physics and Electronic Science, East China Normal University, Shanghai, 200241 China

^*^ Corresponding author.

E-mail address: Jiangzhou83@163.com (J. Peng); and zhang.yong@zs-hosptial.sh.cn (Y Zhang).

^‡^ These authors contributed equally to this work.

**Supplementary Tables**

**Table S1.** Batch-to-batch reproducibility of CSZG. Hydrodynamic diameter, PDI, and zeta potential of three independently prepared CSZG batches. Data are presented as mean ± SD (n = 3 independent batches).

| **Batch** | **Hydrodynamic diameter (nm)** | **PDI** | **Zeta potential (mV)** |
| --- | --- | --- | --- |
| Batch 1 | 180.37 | 0.168 | -8.6640 |
| Batch 2 | 181.25 | 0.162 | -9.0607 |
| Batch 3 | 179.25 | 0.125 | -9.7253 |
| Mean ± SD | 180.29 ± 1.00 | −− | -9.15 ± 0.54 |

**Table S2.** The survival status of BALB/c mice 24 h after the administration of different doses of CSZG. "+" indicated survival and "-" indicated mortality.

| **Dosage of administration**  **(mg/kg)** | **PBS** | **1.25** | **2.5** | **5.0** | **10.0** | **20.0** |
| --- | --- | --- | --- | --- | --- | --- |
| After administration for 0 h | **+** | **+** | **+** | **+** | **+** | **-** |
| After administration for 24 h | **+** | **+** | **+** | **+** | **+** | **-** |

**Supplementary Figures**

**Fig. S1.** (A) Standard curve of the UV-Vis absorbance of Gox. (B) UV-Vis absorbance at 290 nm of CSZG. Data are presented as mean ± SD (n = 3).

**Fig. S2. High-resolution XPS spectra of CSZG.** (A) Cu 2p, (B) Se 3d, and (C) Zn 2p.

**Fig. S3.** **Acid-responsive Cu⁺ release profile of CSZG.** Time-dependent Cu^+^ release from CSZG under pH 6.0 conditions was measured using a neocuproine-based colorimetric assay. Cu^+^ formed a yellow 1:2 complex with neocuproine, and the absorbance was measured at 454 nm. Data are presented as mean ± SD (n = 3).

**Fig. S4. Hemolysis test.** (A) Photographs of hemolysis after incubation with PBS (negative control), H_2_O (positive control), ZIF-8, ZIF-8@GOx, Cu_2-x_Se, CSZ, and CSZG. (B) Quantification of hemolysis rate. Data are presented as mean ± SD (n = 3).

**Fig. S5. Uncropped Western blot images corresponding to Fig. 6A in A549 cells.** Uncropped Western blot images of FDX1, DLAT, NDUFS1, SDHB, and β-actin in A549 cells after treatment with PBS, NIR, ZIF-8, ZIF-8@GOx, CSZ, CSZG, or CSZG + NIR.

**Fig. S6. Uncropped Western blot images corresponding to Fig. 6C in H460 cells.** Uncropped Western blot images of FDX1, DLAT, NDUFS1, SDHB, and β-actin in H460 cells after treatment with PBS, NIR, ZIF-8, ZIF-8@GOx, CSZ, CSZG, or CSZG + NIR.

**Fig. S7. Uncropped Western blot images corresponding to Fig. 6E in A549 cells.** Uncropped Western blot images of FDX1, DLAT, NDUFS1, SDHB, and β-actin in A549 cells from the TTM rescue experiment, including PBS, CSZG + NIR, TTM, and CSZG + NIR + TTM.

**Fig. S8. Uncropped Western blot images corresponding to Fig. 6G in H460 cells.** Uncropped Western blot images of FDX1, DLAT, NDUFS1, SDHB, and β-actin in H460 cells from the TTM rescue experiment, including PBS, CSZG + NIR, TTM, and CSZG + NIR + TTM.

**Fig. S9. Acute toxicity.** (A) Evaluation of the maximum tolerable dose (MTD) of CSZG in healthy BALB/c mice to assess its toxicity. MTD of CSZG in healthy BALB/c mice was determined by administering different doses of CSZG and observing the mortality of the mice 24 h after the treatment. (B) Hematoxylin and eosin (H&E) staining of major organs (heart, liver, spleen, lung, and kidney) collected from mice after being administered different doses of CSZG. Scale bar: 200 μm.

**Fig. S10.** **H&E staining of major organs after ZIF-8 treatment.** Representative histological images of heart, liver, spleen, lung, and kidney from mice treated with different doses of ZIF-8 (2.5, 5, 10, and 20 mg/kg). Scale bar = 100 μm.

**Fig. S11.** (A) Quantification of TUNEL-positive area in tumor sections after different treatments. (B) Quantification of CD31-positive area in tumor sections after different treatments. Data are presented as mean ± SD (n = 3). Statistical significance was determined by one-way ANOVA with Tukey’s post hoc test (**p* < 0.05, ***p* < 0.01, ****p* < 0.001, *****p* < 0.0001; ns, not significant).

**Fig. S12. Flow cytometric analysis of splenic T lymphocytes.** (A) Flow cytometry plots showing CD4^+^ and CD8^+^ T cell populations in the spleens of BALB/c mice receiving PBS, CSZ, CSZ + NIR, CSZG, or CSZG + NIR. (B and C) Quantitative analysis of the relative proportions of (B) CD4^+^ and (C) CD8^+^ T cell populations. Data are presented as mean ± SD (n = 3).

**Fig. S13.** **Flow cytometry gating strategy for the analysis of CD11b^+^ tumor-infiltrating immune cell polarization.** Tumor tissues were processed into single-cell suspensions and analyzed by flow cytometry. Cells were first gated based on FSC-A/SSC-A to exclude debris, followed by singlet selection to exclude doublets and aggregates. CD11b^+^ tumor-infiltrating myeloid/macrophage-enriched cells were then gated, and CD86^+^ and CD206^+^ populations within the CD11b^+^ gate were analyzed using histogram plots.

**Fig. S14.** **Serum biochemical analysis of hepatic and renal function markers after different treatments.** (A–D) Serum biochemical levels of (A) ALT, (B) AST, (C) UREA, and (D) CREA in the eight treatment groups on day 12. Data are presented as mean ± SD (n = 3). Statistical significance was determined by one-way ANOVA with Tukey’s post hoc test (**p* < 0.05, ***p* < 0.01, ****p* < 0.001, *****p* < 0.0001; ns, not significant).

**Fig. S15.** Representative H&E images of the heart, liver, spleen, lung, and kidney collected from mice receiving PBS, CSZ, CSZ + NIR, CSZG, or CSZG + NIR. Scale bar: 100 μm.

**Fig. S16. Hemolysis test of CSZG.** (A) Observation of hemolytic effects of different concentrations of CSZ and CSZG. (B) Quantification of hemolysis rate. Data are presented as mean ± SD (n = 3).
